# Deblender: a semi−/unsupervised multi-operational computational method for complete deconvolution of expression data from heterogeneous samples

**DOI:** 10.1186/s12859-018-2442-5

**Published:** 2018-11-07

**Authors:** Konstantina Dimitrakopoulou, Elisabeth Wik, Lars A. Akslen, Inge Jonassen

**Affiliations:** 1 Centre for Cancer Biomarkers CCBIO, Department of Informatics, University of Bergen, Bergen, Norway; 2 Computational Biology Unit, Department of Informatics, University of Bergen, Bergen, Norway; 3 Centre for Cancer Biomarkers CCBIO, Department of Clinical Medicine, Section for Pathology, University of Bergen, Bergen, Norway; 4 Department of Pathology, Haukeland University Hospital, Bergen, Norway

**Keywords:** Gene expression, Cellular heterogeneity, Deconvolution, Matrix factorization, Particle swarm, Quadratic programming, Clustering, Model selection

## Abstract

**Background:**

Towards discovering robust cancer biomarkers, it is imperative to unravel the cellular heterogeneity of patient samples and comprehend the interactions between cancer cells and the various cell types in the tumor microenvironment. The first generation of ‘partial’ computational deconvolution methods required prior information either on the cell/tissue type proportions or the cell/tissue type-specific expression signatures and the number of involved cell/tissue types. The second generation of ‘complete’ approaches allowed estimating both of the cell/tissue type proportions and cell/tissue type-specific expression profiles directly from the mixed gene expression data, based on known (or automatically identified) cell/tissue type-specific marker genes.

**Results:**

We present Deblender, a flexible complete deconvolution tool operating in semi−/unsupervised mode based on the user’s access to known marker gene lists and information about cell/tissue composition. In case of no prior knowledge, global gene expression variability is used in clustering the mixed data to substitute marker sets with cluster sets. In addition, we integrate a model selection criterion to predict the number of constituent cell/tissue types. Moreover, we provide a tailored algorithmic scheme to estimate mixture proportions for realistic experimental cases where the number of involved cell/tissue types exceeds the number of mixed samples. We assess the performance of Deblender and a set of state-of-the-art existing tools on a comprehensive set of benchmark and patient cancer mixture expression datasets (including TCGA).

**Conclusion:**

Our results corroborate that Deblender can be a valuable tool to improve understanding of gene expression datasets with implications for prediction and clinical utilization. Deblender is implemented in MATLAB and is available from (https://github.com/kondim1983/Deblender/).

**Electronic supplementary material:**

The online version of this article (10.1186/s12859-018-2442-5) contains supplementary material, which is available to authorized users.

## Background

In the era of Systems Medicine, the comprehension of disease etiology and pathogenesis has undergone a paradigm shift with the integration of multiple omics manifestations playing the leading role [1–3]. The impact is more evident in cancer research where integromics approaches have already shown their potential to provide a more effective and accurate means for cancer biomarker discovery [4, 5]. A key component in these studies is the transcriptome data descending from microarrays or RNA sequencing. However, standard approaches for the analyses of expression data are highly affected by the cellular heterogeneity present in tissue samples and the variations in cell type composition [6, 7]. Tumor bulk tissue samples are still often analyzed without considering their complexity and the interactions among the cell types forming the tumor microenvironment [8]. The microenvironment has been suggested to alter under different pathophysiological states, contributing to the comprehension of diverse diseases [9]. Thus, in order to detect the true expression differences related to the different pathophysiological states rather than alterations in cell/tissue type composition, it is imperative to deconvolve the recorded mixed expression measurements into the component expression profiles of each cell/tissue type.

Although experimental approaches like cell sorting, laser-capture microdissection and single cell sequencing can be used to unravel cellular heterogeneity, there is also a growing interest in in silico deconvolution. The advances in this field have shown that computational prediction has its advantages such as low time consumption and the ability to analyze expression responses from multiple cell types simultaneously, and importantly, avoiding experimentally perturbing the samples [10]. The majority of computational deconvolution approaches employ the linearity assumption in which the gene expression level in a mixture of cell populations/tissues is modeled as the sum of gene expression of the constituent cell/tissue types weighted by their proportion in the mixture [6, 11]. Methods for deconvolution can be classified in two major types [12]: (a) *partial* deconvolution methods requiring as input either cell/tissue type-specific expression profiles or mixture proportions [13–18]; (b) *complete* deconvolution methods estimating both the cell/tissue type reference profiles and the proportions directly from the global mixed gene expression data. In the second type, the methods can be further divided into semi-supervised and unsupervised. The first assume that a set of marker genes is given for each cell/tissue type [9, 19] while the latter require no such information. In the latter case, one makes the assumption that the variation in gene expression levels to a large extent is explained by the variation in mixture proportions across samples and marker genes are derived from a set of genes showing high variability in expression across the mixed samples [7, 20, 21].

Computational tools can be further classified based on the type of the gene expression data used as input with most tools being designed for and tested on microarray data and fewer for RNA-Seq data [6]. It has been questioned and analyzed whether methods developed for microarray-based gene expression data can also be applied to RNA-seq data. There are studies stating that there are no confounding factors that make current methods inappropriate for analysis of RNA-Seq data, since they have observed a significantly linear association between RNA concentrations and sequence reads [10, 22], compared to the not-so-linear microarrays [10, 23]. Other studies like Liebner et al. [7] suggest adaptation and incorporation of statistical models appropriate for analysis of RNA-seq data. Recently, for DeconRNASeq [24] it has been shown that the established linear latent model widely used in microarray-based techniques can also be used for deconvolution of data from RNA-Seq applied to mixed samples.

Here, we propose Deblender, a novel complete semi−/unsupervised deconvolution tool for “deblending” heterogeneous microarray and RNA-Seq data. The tool covers many usage scenarios with respect to what information is known, like available marker gene lists, number of constituent cell/tissue types in the mixture, and whether the mixed samples being studied outnumber the cell/tissue types. One feature distinguishing it from other published methods is that it utilizes as information source the global gene expression differences across cell/tissue types directly from the mixed dataset instead of genes with the highest variability often regarded as cell type-specific markers. These differences across cell types have been observed in recent expression studies [25, 26]. Based on this assumption, we employ clustering as means for distinguishing cell/tissue type-specific gene groups and use those (along with their cluster exemplars) to substitute marker genes. Similar ideas have also been explored by an unpublished method, ClusDec R package [27]. In this way we alleviate the need for marker genes known a priori and for setting arbitrary thresholds for detecting genes showing highly variable expression.

In contrast to most other existing methods, Deblender requires no information about the number of cell/tissue types present in the samples under analysis. Similar to Wang et al. [21], we apply an information theoretic model selection criterion based on the Minimum Description Length (MDL) principle. Other information theoretic model selection criteria, like Bayesian and Akaike Information Criterion, and principal component analysis have also been used to estimate number of cell subpopulations in the mixture based on copy number aberrations, DNA methylation or expression data [8, 24, 28]. Furthermore, Deblender is able to analyze datasets where the number of cell/tissue types exceeds the number of samples. Only a few relevant deconvolution methods can be applied to analyze such datasets [7, 21, 29].

The performance of Deblender has been assessed and compared to those of a set of partial and complete state-of-the art deconvolution methods summarized in Table 1. For this, we used several benchmark and patient mixture datasets with known mixture proportions or approximations of those. The results show that Deblender, when executed in complete unsupervised mode, performs *on par* with methods that require additional information to perform deconvolution. Therefore, we believe that Deblender can serve successfully at least as a tool for preprocessing mixed datasets providing an initial approximation of the cell composition. This can be used to seed other experimental or in silico reference-based techniques that may provide a more accurate deconvolution. The use of such techniques can thus be broadened to cases where no external information is available.

**Table 1:** Summary of the features of the proposed Deblender and the methods examined for comparison. With the term ‘input’ we refer to any type of information other than the mixed expression data

	*Classification: Partial or Complete*	*Mathematical/ Statistical method*	*Input: reference/ signature expression data*	*Input: marker gene lists*	*No input*	*Output:* *Cell/tissue type-specific proportions (A)*	*Output:* *Cell/tissue type-specific expression profiles (S)*	*Output: number of cell/tissue types*
MMAD [7]	C/P	Maximum likelihood/ Conjugate gradient	Yes	Yes	Yes^a^	Yes	Yes	No
DSA [9]	C	Quadratic programming	No	Yes	No	Yes	Yes	No
NMF-CELLMIX [12]	C	Non-negative Matrix Factorization	No	Yes	No	Yes	Yes	No
CIBERSORT [16]	P	v-Support Vector Regression	Yes	No	No	Yes	No	No
DeconRNASeq [24]	P	Quadratic programming	Yes	No	No	Yes	No	Yes
Deblender	C	Least Squares/ Quadratic programming/ Non-negative Matrix Factorization/ Unified Particle Swarm Optimization	No	Yes	Yes	Yes	Yes	Yes

^a^MMAD requires as input the number of cell/tissue types

## Results

**Fig. 1:**
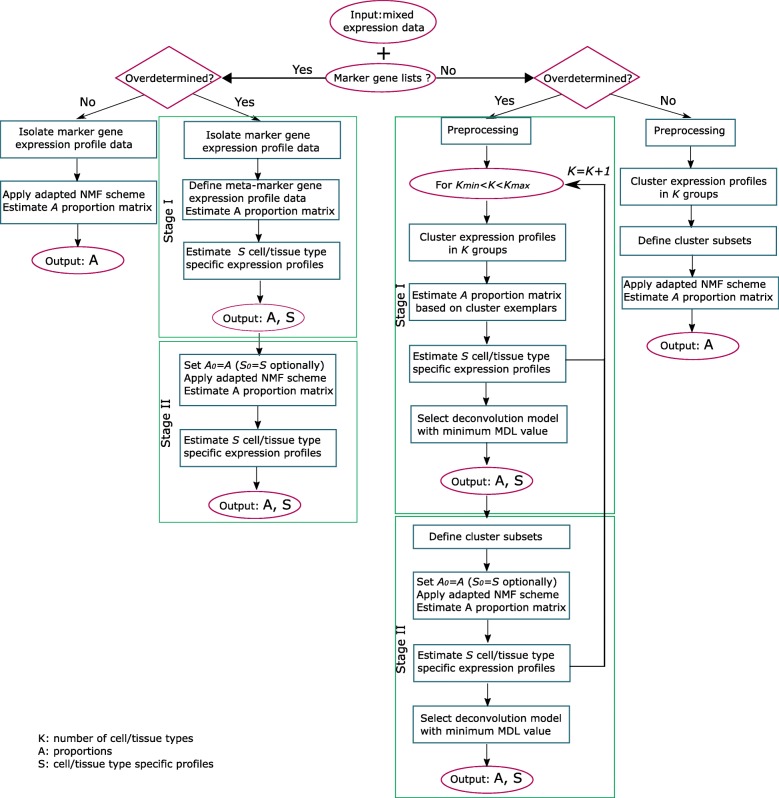
Overview of Deblender. Deblender is a flexible tool operating both on semi- and unsupervised mode based on the availability of marker gene lists. More problem-specific pipelines are also available depending on the number of samples relative to the number of cell/tissue types (under-determined refers to the case where the number of samples is lower than the number of involved cell/tissue types, otherwise over-determined) and on information about the number of participating cell/tissue types

Deblender offers four pipelines (two semi- and two unsupervised) for estimating the mixture proportions and cell/tissue type-specific profiles from mixed microarray or RNA-Seq data (Fig. 1). The appropriate pipeline is determined by the availability of marker gene lists, whether the number of constituent cell/tissue types is assumed to be known, and whether the mixed samples outnumber the cell/tissue types.

To examine the efficiency of Deblender for estimating the mixture proportions, we employed several benchmark mixture datasets (including both microarray and RNA-Seq data) with well-defined cell subpopulations (see Additional file 1) comparing the performance of Deblender with a set of other deconvolution tools. For use in partial and complete semi-supervised methods, we derived marker gene lists or gene expression signatures for the cell/tissues in the datasets, either from the literature [9, 30] or by use of other tools [16, 24]. Also, two patient cancer expression datasets (one microarray and one RNA-Seq) were explored and estimates of cell type proportions obtained using deconvolution approaches were compared to analogous estimates from flow cytometry and histology.

For completeness, we compared Deblender not only to other complete semi-supervised and unsupervised techniques, but also to two robust partial methods, CIBERSORT and DeconRNASeq. To assess the accuracy of each method, we calculated the Root Mean Squared Error (RMSE) and the Pearson correlation coefficient to compare the estimated mixture proportions and the known (or otherwise measured) cell type proportions. RMSE was calculated both based on the full proportion matrices as well as on the proportions of each cell/tissue type separately with the arithmetic mean value reported (mRMSE).

### Estimating mixture proportions in benchmark expression data

All methods were applied to the set of probe/gene expression data that fits to their operational mode (details are provided in Additional file 1). Deblender operates under two algorithmic schemes applied consecutively in two stages (stages I and II). Here, we report primarily the results from stage I (S1) while the result from stages I and II (S1 & S2) is reported in cases where improved performance was achieved. First, Deblender in the unsupervised mode (S1) was tested on all recorded probes/genes (annotated and un-annotated) and the performance in terms of correlation with known mixing proportions ranged in [0.78 − 0.96], while on the preprocessed datasets (i.e., using only annotated probes, one selected per gene identifier) ranged in [0.72 − 0.89] (Additional file 1: Tables S1-S2). Second, we report the estimated proportions based on a ‘default’ filtering setting (retains 53 − 74% of the genes) that performs well across all datasets and is likely to work well on most real datasets. Other settings are described in detail in Additional file 1.

**Fig. 2:**
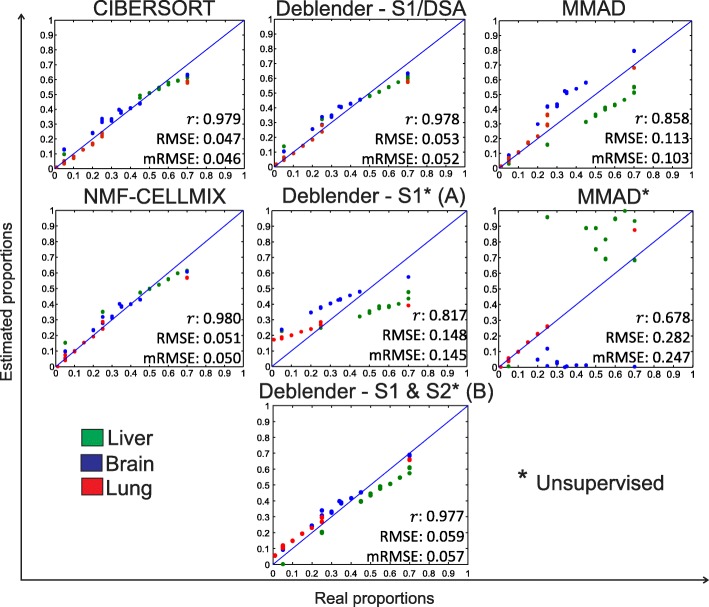
GSE19830 dataset with 33 mixed samples including 3 tissues (brain, liver, lung). Evaluation of methods relative to real mixture proportions based on known markers (partial: CIBERSORT, complete semi-supervised: Deblender, DSA, MMAD, NMF-CELLMIX) or without prior information (complete unsupervised: Deblender*, MMAD*). Deblender* results are reported with (A) default preprocessing – S1 (B) default preprocessing – S1&S2. mRMSE: arithmetic mean of the RMSE calculated for each tissue separately

The GSE19830 microarray dataset includes 33 mixed samples composed of known proportions of pure rat brain, liver and lung tissue. Partial and complete semi-supervised methods were evaluated both on a set of 237 marker probes and a 171 probe signature matrix. Figure 2 summarizes the results obtained on the 237 marker probes. In terms of how accurately they estimate mixture proportions, Deblender in its complete semi-supervised mode and NMF-CELLMIX performed similar to each other and to the partial CIBERSORT algorithm. Notably, in S1 semi-supervised mode, Deblender applies the deconvolution method implemented in DSA tool and thus reproduces the same results therefore both tools are reported. Similar results were obtained when the set of 171 signature probes was examined (see Additional file 1: Table S3). When switching to unsupervised mode, denoted by ‘*’, Deblender* outperformed MMAD* (for other settings see Additional file 1: Tables S4-S7). This dataset serves as good example for exploring the estimation of proportions with varying tissue ratios across samples (a side-by-side comparison of Deblender* estimates relative to real proportions is provided in Additional file 1: Table S8). The performance of Deblender* was high (*r* = 0.89) also when all probes were utilized (without dataset preprocessing - see Additional file 1: Table S1). Similar observations can be drawn for the GSE11058 and GSE19380 benchmark microarray datasets (Additional file 1: Figures S1-S2, Tables S1-S2, S4-S7, S9-S10).

**Fig. 3:**
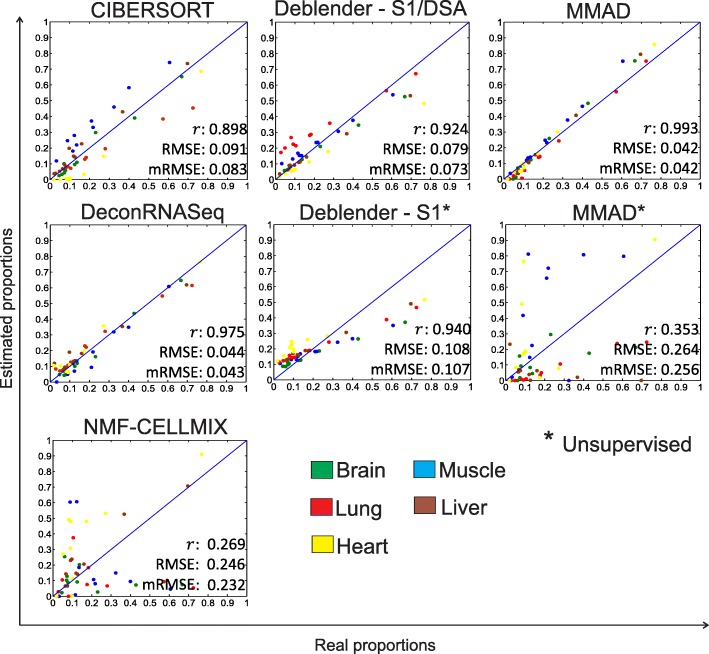
RNA-Seq dataset with 10 mixed samples including 5 tissues (brain, muscle, lung, liver, heart). Evaluation of methods relative to ground truth mixture proportions based on a set of signature genes (partial: CIBERSORT, DeconRNASeq), on markers extracted from the signature set (complete semi-supervised: Deblender, DSA, MMAD, NMF-CELLMIX) or without prior information (complete unsupervised: Deblender*, MMAD*). Deblender* result is reported with default preprocessing – S1

Next, we analyzed an RNA-seq dataset which includes 10 mixed samples composed of human brain, muscle, lung, liver and heart tissue, in known proportions [24]. A set of 1520 signature genes was extracted by DeconRNASeq. CIBERSORT and DeconRNASeq were run with the full signature matrix while the complete semi-supervised methods were run with the 5-fold differentially expressed genes (i.e., genes expressed at least 5-fold higher in the respective cell type relative to any of the other cell types). The results are in agreement with those for the other datasets (Fig. 3). Complete semi-supervised techniques like Deblender/DSA and MMAD showed high performance and in some cases even higher than partial techniques. When switching to complete unsupervised mode, Deblender* outperformed MMAD* (see also Additional file 1: Tables S2, S4, S6, S7). A side-by-side comparison of Deblender* estimates against real proportions is provided (Additional file 1: Table S11). Moreover, we checked how common preprocessing steps in RNA-seq analysis – such as adding a pseudo-count offset to avoid zero values in downstream analyses – affected the performance of Deblender* and MMAD*. We checked two offsets, 0.0001 and 1. MMAD* did not perform well with the offset of 0.0001 since it affected the identification of highly variable genes by causing an inflation of the variance of low abundance genes after log transformation. When tested with an offset of 1, MMAD* improved and performed better than Deblender* in certain parameter settings whereas Deblender* preserved its good performance as with the offset of 0.0001 (see Additional file 1: Table S12).

The unsupervised methods were also run with different parameters, dataset filtering settings and with noise added (see Additional file 1: Tables S4-S7, S10, S13, S14).

### Estimating mixture proportions in under-determined cases based on benchmark expression data

**Fig. 4:**
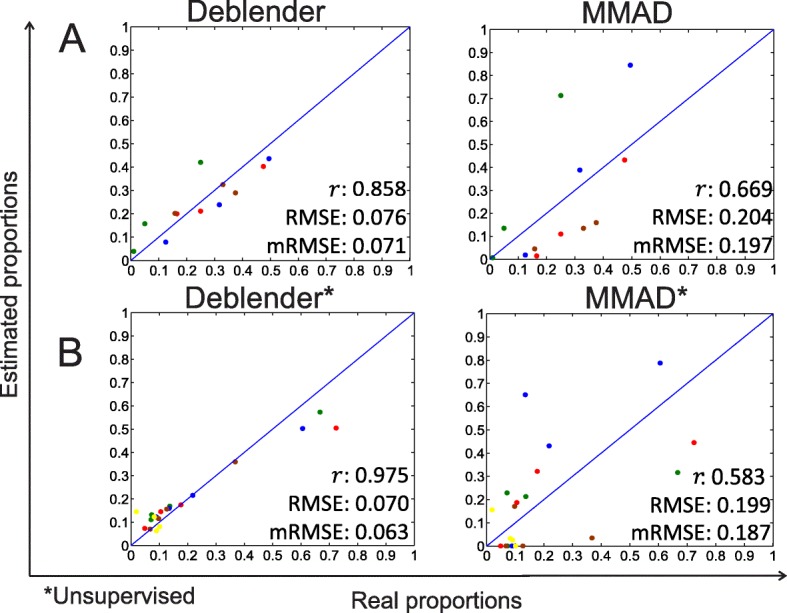
Evaluation of Deblender and MMAD (A) on a subset dataset extracted from GSE11058 with 3 samples including 4 cell types (semi-supervised mode) and (B) a subset dataset extracted from the RNA-Seq with 4 samples including 5 tissues (unsupervised mode)

We examined the under-determined case relevant for semi-supervised and unsupervised methods where the number of samples is less than the number of involved cell/tissue types. We evaluated Deblender and MMAD on a subset dataset from GSE11058 (i.e., 3 samples containing 4 cell types) and on a subset dataset from the RNA-Seq dataset (i.e., 4 samples containing 5 tissues). For the first dataset, we checked the performance based on known marker genes, while in the latter we checked the unsupervised mode. For MMAD* we applied default percentile (see also Additional file 1: Table S6). Deblender outperformed MMAD in both cases (Fig. 4).

### Estimating the number of cell/tissue types in benchmark expression datasets

**Fig. 5:**
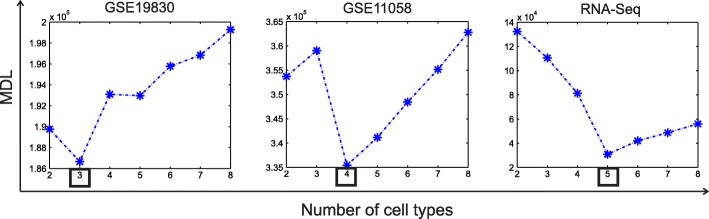
Performance evaluation of Minimum Description Length (MDL) criterion for detecting the number of cell/tissue types present in three mixture datasets. The boxes mark the true number of involved cell/tissue types

We evaluated the efficiency of MDL criterion integrated in Deblender* for estimating the number of cell/tissue types present in the mixture. For this, we selected the GSE19830, GSE11058 and RNA-Seq datasets in which all cell/tissue types are present in all mixed samples. For all datasets we applied the unsupervised mode S1&S2 and recorded the MDL value with *k* ranging from 2 to 8 after filtering the 5% of genes with the lowest expression vector norm and the 5% of genes with the highest expression vector norm. We also used a cutoff of CV ≥ 0.4, since we observed that highly variable genes improve MDL computation. As seen in Fig. 5, for all datasets the minimum of the MDL curve predicted successfully the correct number of cell/tissue types.

### Estimating mixture proportions in patient cancer expression data

**Fig. 6:**
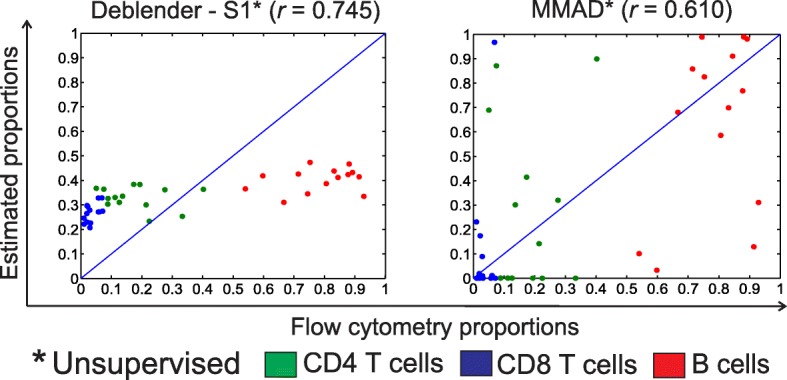
GSE65135 dataset with 14 mixed samples including 3 cell types (CD4 T cells, CD8 T cells and B cells). Evaluation of the unsupervised mode of Deblender* (default preprocessing – S1) and MMAD* (default percentile) relative to flow cytometry proportions. Pearson correlation (*r*) results per cell type, Deblender*: CD4 T cells: -0.178, CD8 T cells: 0.535, B cells: 0.266, MMAD: CD4 T cells: 0.099, CD8 T cells: 0.301, B cells: 0.352

We evaluated Deblender* and MMAD* on patient cancer expression datasets for which only estimates of the real proportions are available. First, we examined GSE65135 microarray dataset which contains 14 follicular lymphoma samples consisting of CD4+ T cells, CD8+ T cells and B cells, with proportions estimated based on flow cytometry data. As shown in Fig. 6, Deblender* (default setting – S1) performed better in terms of correlation with the flow cytometry proportions than did MMAD* (default percentile, see also Additional file 1: Tables S6 and S7, Figure S3 and Additional file 2). Further, the MDL estimation indicated *K* = 2 as the number of involved cell types with *K* = 3 being close to this minimal value (Additional file 1: Figure S4).

We also evaluated the performance of Deblender* and MMAD* on the TCGA RNA-Seq data of 1093 breast cancer primary solid tumor samples [31]. We used a simplified model with three major tissue components for which histological estimates are available for the main types of tissue components recognized on the tissue slides (normal, stromal and tumor). Of note, the MDL estimation of Deblender showed that the number of involved tissue components ranges between 15 and 26 (Additional file 1: Figure S4) and this observation accords well with the prediction of 23 cell types in the TCGA samples by relevant study [8]. Deblender* and MMAD* were tested both with their ‘default’ settings (as used for the benchmark datasets), which include in the analysis many of the lowly expressed genes, but also with a ‘customized’ setting that discards them. Finally, ~76% of the complete gene set was retained and for Deblender* no other filtering was applied. We performed pathway enrichment analysis on the three clusters identified by Deblender* in the customized setting and checked the enriched Gene Ontology (GO) categories and Kyoto Encyclopedia of Genes and Genomes (KEGG) pathways. The enriched terms were matched to each tissue component after considering the cluster order configuration that led to the highest correlation with the known histology estimates (see Additional file 3). GO categories reflecting immune response activity were top ranked amongst the categories enriched in the cluster corresponding to ‘Normal’. GO terms reflecting various metabolic processes were significantly enriched in the cluster that corresponded to ‘Stromal’, as also reflected by the enriched KEGG pathway terms. GO terms reflecting metabolism at different levels were also enriched in the cluster that corresponded to ‘Cancer’. Further, various gene sets reflecting different cancer associated pathways and insulin related signaling were enriched in the ‘Cancer’ cluster.

**Fig. 7:**
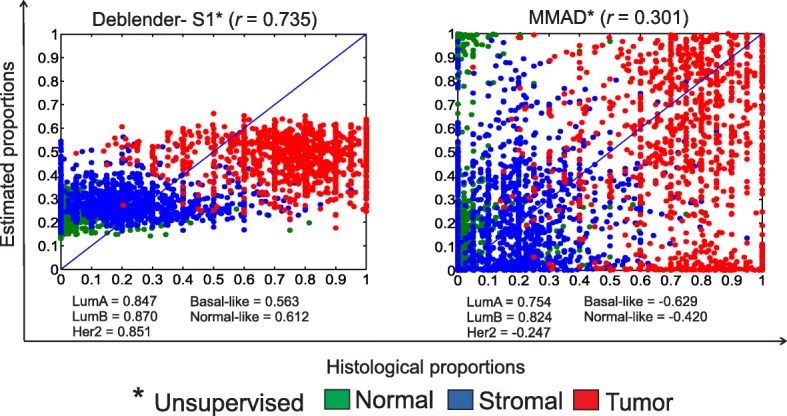
TCGA breast cancer RNA-Seq dataset with 1093 primary tumor mixed samples including 3 defined tissue components (normal, stromal, tumor). Evaluation of the unsupervised mode of Deblender* (customized setting – S1) and MMAD* methods (customized setting – default percentile) relative to histological estimates. Correlation is also reported for subsets of samples with known molecular subtype. Pearson correlation (*r*) results per tissue component, Deblender*: Normal: − 0.051, Stromal: − 0.022, Tumor: -0.061, MMAD: Normal: − 0.041, Stromal: 0.071, Tumor: − 0.026

In Fig. 7, we show the results based on the ‘customized’ setting where Deblender* (S1) performed better than MMAD* (default percentile) in terms of correlation with the histological estimates (see also Additional file 1: Tables S6 and S7 and Additional file 2). Similar were the results for Deblender* when the ‘default’ setting was applied (*r* = 0.74). Notably, when looking each tissue component independently, no significant correlation was found between real proportions and MMAD* and Deblender* estimates (Additional file 1: Figure S5), respectively. The overall better correlation of Deblender* reflects its ability to better recover for each sample the relative abundance of each tissue component. The Deblender* mean proportion value of the tumor component was lower (S1:*μ* = 0.48, *σ* = 0.08) than the respective histology-based estimates (*μ* = 0.74, *σ* = 0.18). When zooming into subsets of samples with known molecular subtype (Luminal A, Luminal B, Basal-like, Her2-enriched, Normal breast-like), Deblender* (S1) showed higher performance for Basal-like, Her2-enriched and Normal breast-like groups.

Since Deblender* used the full set of expression data to assign mixture proportions, its results may depend strongly on the set of samples included in the analysis. To assess this dependence, we also applied Deblender* on a bigger dataset including, in addition to the 1093 primary tumor samples, also 7 metastasis and 112 normal samples. The tissue composition is assumed to be highly different between primary tumor, metastasis and normal samples. We observed that only when applying the customized setting and *CV* ≥ 1 (analyzing ~23% of the complete gene set), the overall correlation of primary tumor samples relative to known mixture proportions decreased (S1: *r* = 0.54) but the normal samples achieved higher mean proportion value for the ‘normal’ tissue component (S1: *μ* = 0.54, *σ* = 0.10) relative to the mean value of the primary and metastasis tumor samples respectively (S1: *μ* = 0.19, *σ* = 0.06). Also, the small cohort of metastasis samples displayed mean values for all components similar to those observed in the primary tumor samples.

We further evaluated the agreement of Deblender* estimated proportions relative to the tumor purity estimates (i.e., the proportion of cancer cells in the mixture) assigned by other relevant methods that used gene expression or other TCGA genomic data such as somatic copy-number variation, somatic mutations and DNA methylation. For this, we downloaded the results from the different methods from Aran et al. [32], where a systematic analysis of a set of methods (ESTIMATE, ABSOLUTE, LUMP, IHC) as well as an additional consensus method (CPE) is presented. In this case, Deblender* was run in the cohort of primary tumor samples both with three tissue components and in a constrained fashion with two – corresponding to tumor and non-tumor component. We ran Deblender* using three tissue types (S1) as aforementioned and also with two tissue types using two different settings (S1, setting 1: no filtering, setting 2: *CV* ≥ 3). We checked across all samples the correlation of Deblender* tumor purity estimates relative to those estimated by each method and found in general a low but positive correlation with respect to the consensus method (CPE) (S1: *r* = 0.24, for the three-tissue-component model, *r* = 0.15 and *r* = 0.35, for the two-tissue-component model) (see also Additional file 1: Figures S6-S11). However, when restricting our analysis to the set of samples where Deblender* proportions deviated in the range [−0.2, +0.2] from CPE results, we found moderate or high correlations (S1: *r* = 0.56, *r* = 0.80 for 64% and 43% of the samples) in the two-tissue-component model. In the pairwise comparisons, Deblender* results correlated more with ABSOLUTE and LUMP scores.

### Runtime

We recorded the elapsed time (tic-toc result in Matlab) of Deblender* unsupervised mode for calculating mixture proportions (S1 & S2) and MDL in two benchmark datasets (GSE19830 and RNA-Seq) with the respective default settings (Additional file 1: Figure S12). All tests were run on a 2.40 GHz Intel Core i7 with 7.65G RAM running Windows 7.

## Discussion

In silico deconvolution modeling started with partial approaches requiring as available, either the cell/tissue type-specific expression profiles or mixture proportions, but gradually complete semi-supervised and more importantly unsupervised methods have gained ground. The unsupervised ones showed that representative profiles - sufficient to decompose the signal - can be extracted directly from the mixed data alleviating the need for additional experiments or borrowing reference data from other sources.

In this work, we present Deblender, a new flexible complete deconvolution tool with semi- and unsupervised operational modes which integrates features introduced by recent approaches [7, 9, 12, 19, 21, 33] and proposes several novel concepts. First, Deblender adopts the deconvolution model and constraints proposed by others [9, 12, 19], based solely on marker gene lists, and extends this concept into unsupervised by employing a flexible assumption about the gene cell/tissue type-specific expression. In particular, we assume that many genes show differences in terms of relative expression among the different cell/tissue types [25]. Lately, single-cell sequencing studies like Dueck et al. [26] have shown that gene expression differs globally across tissues in terms of the number of genes expressed, the average expression pattern and the within-cell-type variation patterns. Although each cell type exhibits a characteristic transcriptome profile enriched in marker genes, the marker gene expression is rarely if ever limited to the relevant cell type [26]. Moreover, some marker genes show significant variability within the relevant cell type, indicating that these genes are not sufficient to determine the cellular phenotype [26]. Under this notion, we apply clustering to identify groups of genes prone to be more expressed in a specific cell/tissue type and employ those clusters (along with their cluster exemplars) with the constraints others use for the marker sets. In this way, we overcome all the arbitrary cutoffs/criteria that most partial and complete methods face when selecting the small cohort of signature/marker genes. The cluster-based concept was adapted to two different algorithmic approaches. First, we adapted the unsupervised algorithm of Zhong et al. [9] for estimating mixture proportions after isolating the marker mixed gene expression profiles and subsequently estimating cell/tissue type-specific expression profiles for all recorded genes based on the estimated proportion result. Second, we adapted the Non-negative Matrix Factorization scheme of Gaujoux and Seoighe [12, 19] which estimates mixture proportions and cell/tissue type-specific profiles based on all genes with the marker genes constrained to express only in the relevant tissue/cell type. Deblender runs primarily the first algorithm (referred to as S1) which we have found to work well on a set of benchmark datasets. The results of this algorithm can further be used to initialize the second (referred to as S2). We suggest using S1&S2 after evaluating the clustering result where cluster exemplars that do not differentiate well from each other might not serve as good candidates for deconvolution with S1. Third, we extend our unsupervised method by incorporating an information theoretic criterion to predict the number of cell/tissue types. In the work of Wang et al. [21], this criterion evaluated the number of cell/tissue types based on predicted small-sized marker gene sets. Here, we show that this criterion can perform equally well in our proposed cluster-based approach. Finally, we introduce an adapted Non-negative Matrix Factorization (NMF) scheme to deal with the challenging under-determined cases for proportion estimation in semi- /unsupervised mode, i.e., cases where the number of cell/tissue types exceeds the number of samples in the dataset.

We assessed the performance of Deblender on a set of benchmark datasets where the real proportions of cell/tissue types are available and cancer patient datasets where only flow cytometry or histological estimates are available. For comparison, we recruited several partial and complete state-of-the-art methods (see Additional file 1 for short description). The results on benchmark datasets showed that both the semi-supervised and the unsupervised mode of Deblender performed in both the over- and under-determined cases similarly to the comparative reference-based methods included in the analyses. At this point it is worth commenting that the extra information included in the partial deconvolution methods as compared to the complete semi-supervised ones (that is reference expression profiles in partial methods compared to marker gene sets for the semi-supervised ones) is not always translated into better performance. With respect to unsupervised mode, we showed that cluster sets (and their cluster exemplars) descending from the mixed gene expression dataset can serve as a successful alternative to externally defined lists of marker genes. This indicates that large part of the transcriptome carries considerable cell/tissue type-specific information, i.e., many genes have cell/tissue-type dependent expression levels. Therefore, clustering using expression across most genes can lead to successful signal decomposition. We have also seen that in some cases, it is beneficial to include only the highly variable genes in the clustering. This may depend on which cell/tissue types are included and also on how much cell type proportions vary among the samples analyzed. If all samples have highly similar cell/tissue-type proportions, the approach will not work. Similarly, the method is not appropriate for cases where the sample cohort includes both mixture and one-cell-type samples due to the clustering involved. Moreover, the results showed that Deblender* is applicable on RNA-Seq data and overcomes preprocessing challenges that one faces when dealing with RNA-seq data, e.g., choosing the right pseudo-count offset prior to log transformation. However, for some of the benchmark datasets and also on both real datasets Deblender produced estimates that considerably deviated from known (or otherwise approximated) proportions. This can be attributed to (a) the clustering which may determine cluster exemplars that most likely do not represent well distinct cell/tissue types (each cluster having contributions from multiple underlying components), as compared to marker genes, resulting so in higher errors in the estimates of cell type proportions, (b) deconvolution results are affected by the inherent measurement noise, biological variability and the fact that the mixed data we have available has been processed and normalized (e.g., quantile normalized) as described in the original articles. The effect of data processing is debated in the literature, and it has been argued that the choice of normalization procedures affects the deconvolution results [34, 35], (c) the multicollinearity issue of having correlated cell types in the mixture [34] and (d) the fact that the flow cytometry and histological estimations in real datasets are similarly an approximation of the real proportions.

Moving forward, Deblender* performed better than MMAD* in the cancer patient expression datasets. The improved performance was more evident in the TCGA breast cancer RNA-Seq dataset, where we checked independently the sets of samples with known molecular subtype identities, although deconvolution was realized on the full set of samples irrespective of this information. We observed the greatest differences for the Basal-like, Her2-enriched and Normal breast-like subtypes whereas similar performance was achieved for Luminal A and Luminal B. In our simplified model with three tissue types for the TCGA dataset, we observed that the mean proportion value for the tumor component was lower (S1:*μ* = 0.48, *σ* = 0.08) compared to the mean estimated by histology (*μ* = 0.74, *σ* = 0.18). However, this difference may have other reasons. First, there is more than one cell type in each tissue component, and some of the signals from the tumor cell component might resemble signaling from normal epithelial cells or the stromal tissue. Second, we applied clustering with the number of clusters being lower than the actual number of tissue components [8]. In this way, the identified cluster exemplars most likely do not represent well distinct cell/tissue components (each cluster having contributions from multiple underlying components), thus affecting deconvolution efficiency. Also, immune cells might be dispersed in the different components, complicating the differentiation of signals between the tissue components. Lately, in the study of Onuchik et al. [8] the intra-tumoral heterogeneity was explored by deconvolving in silico the methylation data of mixed samples into five different cancer epithelial cell types along with one normal epithelial, one immune and one stromal tissue. Third, differences between transcriptome-based and pathology-based estimates have been reported [36]. Here, they hypothesized that reasons for the differences include interobserver bias and differences between the tissue sections of the samples examined and those used for nucleic acid extraction [29]. Further, we checked the performance of Deblender on a cohort of samples with increased biological variability in the inherent component profiles including, in addition to primary tumor samples, metastasis and normal samples. Our unsupervised analysis indicates that lowly or moderately variable genes can reveal more information for the samples with increased heterogeneity, like primary tumor and metastasis samples. However, it is the subset of highly variable/marker genes that can assist in more accurate proportion estimation of samples with one or few tissue components involved, like the normal samples.

Also, we showed in the TCGA breast cancer data that the tumor proportions estimated by Deblender* in a two-tissue-component model (tumor/non-tumor) using 76% of the complete gene set correlated well (S1: Pearson’s *r* = 0.56), for a good proportion of the samples (~64%), with the consensus tumor proportions descending from methods that use genomic information other than gene expression, such as somatic copy-number variation, somatic mutations and DNA methylation [32]. A confounding reason behind comparisons is the fact that Deblender* uses expression information for the whole set of samples before assigning mixture proportions to each sample. Moreover, in this first round of analysis we did not consider the intrinsic molecular subtypes [37] and assumed simplistically that all samples share common tumor and non-tumor profiles. On the other side, the ESTIMATE method [36], which is the method conceptually closest to Deblender, uses reference gene expression data to assign tumor purity in each sample independently. Furthermore, with respect to ABSOLUTE which uses copy number data [38], it has already been commented by Wang et al. [20] that such values are generally ‘static’, while gene expression values are intrinsically ‘dynamic’, a realistic assumption especially in cases where the samples are pooled during dynamic complex diseases like cancer. Also the technical variability, i.e., noise, is significantly different between copy number and gene expression signals.

## Conclusions

The Deblender tool represents a methodological framework with a broad repertoire of operational modes for datasets with limited or no access to reference cell/tissue type-specific expression data, marker gene lists and information about the cell/tissue type composition. As such, we believe that it will be of particular interest to studies taking place in a realistic setting, i.e., highly complex tissues and small patient cohorts. Deblender extracts information from the whole transcriptome rather than searching features serving as hallmarks of marker genes. As such, Deblender is a good candidate tool for providing an initial virtual decomposition of the tumor and its microenvironment, situation where the definition of marker genes is not straightforward.

The increasing number of published partial and complete semi-supervised computational deconvolution methods and their recent achievements as predictive and clinical tools [16] have indicated to a great extent that in silico decomposition has great explanatory power and can serve as an appealing low-cost alternative to physical cell separation techniques. Thus, it can facilitate a more extensive adoption of microarray/RNA-seq technology across the continuum from prevention to detection, staging, prognostication and treatment development in complex diseases. Nevertheless, our notion is that this next generation of complete unsupervised techniques - like Deblender - will be a step towards integrating such tools into clinical decision pipelines and allowing researchers to re−/meta-analyze public transcriptome data in a manner that demands little time and performs in a robust fashion. In this way, researchers will gain increased insight into the true disease-dependent expression and mixture proportion differences and be able to generalize results across studies.

## Methods

### Linear model on gene expression deconvolution

We adopt the linear model described in several deconvolution algorithms [9, 12, 19]. Let S be a n × k cell/tissue-type-specific gene expression matrix that contains k cell/tissue types and n genes, A be a k × p matrix with each column representing the mixture proportions of k cell/tissue types in each sample, and X be a n × p mixed expression matrix with n genes and p samples (containing the measured gene expression levels for the mixed samples). The mixing process can be described in terms of matrix notation as:

1 $$ X= SA $$

In *complete* deconvolution both S and A are unknown. In our approach, first the proportion matrix A is calculated based either on marker gene lists (semi-supervised) or cluster sets (unsupervised mode). Then, we estimate the cell/tissue type-specific expression values for each gene independently to finally formulate the whole S cell/tissue type-specific gene expression profile matrix.

### Estimating mixture proportions

Deblender employs the assumption that a subset of genes called markers, i.e., those that are highly expressed in a specific cell/tissue type and lowly expressed in all other cell/tissue types known to be included in the mixture, are appropriate to predict mixture proportions (semi-supervised mode) [9, 12, 19]. In case this information is not available, we extend these methods and apply clustering on the gene expression dataset and subsequently use representative profiles – cluster exemplars – as an alternative. Deblender operates in two stages: in stage I (S1), the proportions are estimated using an approach based on that described by Zhong et al. [9] and in stage II (S2), the proportions are calculated using the method proposed by Gaujoux and Seoighe [12, 19] after using as initialization (in one of many replicated runs of stage II) the proportion matrix (optionally also the cell/tissue type-specific expression profiles based on this proportion result) produced in S1.

Stage I: We first assume S_m_, a m × k matrix representing for each of the k cell/tissue types a set of marker genes (or a cluster) highly expressed in a respective cell/tissue type and zero in the remaining cell/tissue types.

2 $$ {X}_m={S}_mA $$

We simplify further the model (eq. 3) and assume that a meta-marker average expression profile for each set (or cluster exemplar) exists and formulate ${\overset{\sim }{\mathrm{S}}}_{\mathrm{m}}$ (eq.4) as follows:

3 $$ {S}_m=\left(\begin{array}{l}{g}_{11}\kern2em 0\kern1.74em \dots \kern1.98em 0\\ {}{g}_{21}\kern2em 0\kern1.74em \dots \kern1.99em 0\\ {}\kern0.28em 0\kern2.1em {g}_{32}\kern.85em \dots \kern2em 0\\ {}\kern0.28em 0\kern2.12em {g}_{42}\kern1.45em \dots \kern2em 0\\ {}\kern0.28em 0\kern2.12em {g}_{52}\kern1.5em \dots \kern1.9em 0\\ {}\kern0.28em 0\kern2.55em 0\kern1.62em \ddots \kern1.9em 0\\ {}\kern0.28em 0\kern2.57em 0\kern1.8em \dots \kern1.9em {g}_{mk}\end{array}\right) $$

4 $$ \overset{\sim }{S_m}=\left(\begin{array}{l}{m}_1\kern1.12em 0\kern1em \dots \kern1.12em 0\\ {}\;0\kern1.5em {m}_{2\kern0.5em }\dots \kern1.1em 0\\ {}\;0\kern1.72em 0\kern.82em \ddots \kern1.13em 0\\ {}\;0\kern1.72em 0\kern0.98em \dots \kern1.13em {m}_k\end{array}\right) $$

Similar to${\overset{\sim }{\mathrm{S}}}_{\mathrm{m}}$, we create the ${\overset{\sim }{\mathrm{X}}}_{\mathrm{m}}$ of the mixed data by taking for each set the average of the mixed expression profiles of the respective marker genes (or cluster exemplars) and then eq. 2 becomes:

5 $$ {\tilde{X}}_m={\tilde{S}}_mA $$

Since ${\overset{\sim }{S}}_m$ is diagonal matrix, we can multiple both sides with ${\overset{\sim }{S}}_m^{-1}$ and obtain:

6 $$ \overset{\sim }{S_m^{-1}}\kern0.24em \overset{\sim }{X_m}=A $$

Based on the model constraint that the sum of proportions for each sample sums to 1, a new system of equations is formed with k unknown variables, i.e., the diagonal elements of ${\overset{\sim }{\mathrm{S}}}_{\mathrm{m}}^{-1}$:

7 $$ \sum \limits_{i=1}^k{\left(\overset{\sim }{S_m^{-1}}{X}_m\right)}_{ij}=1 $$

When the number of mixed samples is greater than the number of cell/tissue types involved, i.e., when *p* > *k*, we can solve the overdetermined system of equations (eq. 7) with *k* unknown parameters, non-negative constraints and as objective function the minimization of the squared norm of the residual. After estimating the unknown variables of eq. 7 we can return to eq. 6 and estimate the A mixture proportion matrix. Throughout all operation stages of Deblender, in case the sum of estimated proportions for a given sample does not sum to 1, the proportions are scaled accordingly before subsequent analysis.

Stage II: In this stage, we adopt a Nonnegative Matrix Factorization scheme proposed for describing the deconvolution model based on marker genes [12, 19]. The general algorithmic structure of NMF approaches is given a non-negative n × p matrix X, to find, after minimizing common objective functions like the Frobenius norm or the Kullback-Leibler divergence, an approximation

8 $$ X\approx WH $$

where W (i.e., basis components), H (i.e., mixture coefficients) are n × r and r × p non-negative matrices, respectively, and the factorization rank r is often such that r <  < min(n, p).This can be adapted to the deconvolution model if the columns of *H* are constrained to sum to one. *X* represents the mixed gene expression matrix, the columns of the matrix *W* (i.e., *S*) correspond to the cell/tissue type-specific expression profiles and each column of the matrix *H* (i.e., *A*) provides the sample-specific mixture proportions. In the work of Gaujoux and Seoighe [19], more constraints were imposed on the cell/tissue type-specific expression matrix *S* that refer to the marker genes. They demand both during initialization and in each of the iterations of the algorithm, that the rows of *S* that correspond to the markers of each cell/tissue type to be zero-valued in the irrelevant cell/tissue types. During each iteration, the remaining non-zero values of *S* across all genes are updated. In Deblender, in an analogous way we adapt the multiplicative update algorithm (‘nnmf’ Matlab function [39]). In the unsupervised mode, we employ cluster sets instead of marker sets. Specifically, for each cluster set we choose the *n* % (user-defined) of genes closest to exemplars and use these cluster subsets likewise with markers. Then, the NMF-adapted process can be run either on the complete gene set of the mixture dataset (pseudocode is available in Additional file 1) where only the cluster subsets are treated as markers or on the set that includes only genes belonging to the cluster subsets. In both cases the rows of *S* that correspond to each cluster subset will be zero-valued in the irrelevant cell/tissue types throughout the NMF iterations. Of note, as initialization *A*_0_, in one of the replicated experimental runs, the proportion matrix *A* obtained in stage I (S1) is used (optionally *S*_0 _can be the *S* produced based on the proportion result of S1) and the objective function to be minimized is the root-mean-squared residual between *X* and *S* ∗ *A* (as in ‘nnmf’ Matlab function). Similar to [19], we apply the sum-to-one constraint on the final *A* result.

### Estimating cell/tissue type-specific expression profiles

Once the A mixture proportion matrix is known (either from S1 or S1&S2), one can solve the system of equations independently for each gene *i*, as proposed in Zhong et al. [9], and calculate the S_i_ by minimizing:

$$ {\left\Vert x-{AS}_i\right\Vert}^2,{S}_i\prec ub\kern0.5em and\kern0.5em {S}_i\succ 1b $$

where *ub* and *lb* set the constraint that the resulting values are within the boundaries set by the maximum and minimum measurable gene expression levels. Based on this least squares optimization, the average gene expression values of each cell/tissue type are estimated. Similar to Onuchik et al. [8], we extend this so that one can also calculate in this multiple linear regression problem the standard error (SE) for each of the coefficients (i.e., expression level of each gene *i*) in each cell/tissue type *j*:

9 $$ SE\left\{{S}_{i,j}\right\}=\sqrt{{{\left[{MSE}_i\left(A{A}^t\right)\right]}^{-1}}_{i,j}} $$

where *MSE*_*i*_ (mean squared error for gene *i*):

10 $$ {MSE}_i=\frac{\sum \limits_{j=1}^k{R}_{i,j}^2}{p-k} $$

and *p* is the number of samples, *k* the number of cell/tissue types and *R* the residual matrix (*R* = *X* − *SA*).

### Unsupervised mode

#### Data preprocess

Deblender applies deconvolution in the linear space, as established by others [9, 40]. As such, data need to be raw normalized values (without log transformation). Also, as preprocessing step prior applying Deblender, we propose filtering of un-annotated probes and in case of multiple probes per gene identifier (e.g., Entrez ID) selecting the probe with the highest variance across dataset. Deblender applies further filtering steps that have been adopted by other similar approach [21]. The user can first define the percentage cutoffs to filter genes with high (outliers) and/or low (noise) expression vector norms. Subsequently, genes can be further filtered by setting a threshold on the coefficient of variation (CV), defined as the ratio of the standard deviation of the raw gene expression profile to the mean of the raw gene expression profile.

#### Clustering

The user can choose between k-means or k-medoids algorithms for clustering with ‘correlation’ as distance function in order to assign genes to a putative cell type. Data prior to clustering can be either in linear or log scale; we observed that the latter leads to increased performance in some cases. Regardless of the cluster option setting, the cluster exemplars used in S1 are defined in linear scale and can represent for k-means the average mean profile of the cluster members and for k-medoids either the medoid or the average mean profile of the cluster members. In S2, in order to define the cluster subsets, the genes closest to the centroids/medoids (exemplars), as defined in the respective cluster option setting, are considered. In the ‘kmeans’ Matlab function [41], setup parameters like ‘OnlinePhase’ have been turned on to guarantee that the provided solution is a local minimum of the distance criterion. Also, the ‘Replicates’ parameter, i.e., the number of times to repeat clustering using new initial cluster centroid positions, has been set to 500 to achieve optimal performance and stability. We recorded (100 runs) the clustering objective function value as well as the produced proportions in multiple datasets (unsupervised mode – S1) and observed no significant fluctuations. Also, with respect to k-medoids (‘kmedoids’ Matlab function [42]), we chose the ‘large’ algorithm [43] (similar to k-means) due to the large size of the datasets.

#### Cell/tissue type identity

When Deblender operates on unsupervised mode, there is no prior information in which distinct cell/tissue type each cluster exemplar should be assigned to. With respect to benchmark datasets, we checked all possible combinations and preserved the result giving the highest correlation when comparing estimated with real proportions. We validated this concept based on the reference cell/tissue type-specific profiles of the benchmark datasets and observed that in most cases the best order configuration was the one in which the majority of genes of a given cluster showed maximal expression in the assigned cell/tissue type.

#### Model selection

In case where the user has no knowledge about the number of cell/tissue types involved in the mixture, Deblender can automatically estimate, in the unsupervised mode, the number *k* of cell/tissue types present in the mixture. For this, we employ the Minimum Description Length (MDL) information criterion as recently proposed by others for use in expression deconvolution [21]. In particular, Deblender is applied multiple times with a different *k* each time as candidate and the model that explains the observed data, while avoiding unnecessarily complex models, is preferred. The formula of MDL is defined as:

11 $$ MDL(k)=-\log \left(\lambda \left({X}_m|\theta (k)\right)\right)+\frac{\left(k-1\right)p}{2}\log (m)+\frac{km}{2}\log (p) $$

where the first part refers to the joint likelihood function with *X*_*m*_ being the mixed profiles of the genes after filtering process and *θ*(*k*) the set of freely adjustable parameters in the model. The remaining part is the penalty function where in case of *A* proportion matrix calculation for a given sample *m* genes participate, thus in total contributing (*k* − 1)*p* log(*m*)/2 bits, while in the case of *S*_*m*_ calculation *p* scalar entries for a given gene are used, thus contributing in total *k m* log(*p*)/2 bits.

### Estimating proportions in under-determined cases

In case the number of cell/tissue types is higher than the number of available mixed samples, Deblender offers the option (when the number of samples is ≥2) to apply an adapted NMF scheme to approximate the proportions based on available marker gene lists (semi-supervised) or cluster sets (unsupervised). More specifically, we solve the system of eq. 2 after decomposing it into *k* subsystems, i.e., subsets of equation systems, each one corresponding to a cell/tissue type-specific marker gene or cluster subset. The cluster subsets are defined after clustering the preprocessed dataset in *k* cluster sets and retaining for each cluster set *n*% of genes that are closest to the cluster exemplar.

Zooming into each subsystem (eq. 8), *X* is a c × p mixed expression matrix with c cell-specific marker or cluster subset genes and p samples, *W* a c × 1 vector and *H* a 1 × p vector representing the cell/tissue type-specific *S* and *A* vectors (pseudocode and extended description about the formulation of the subsystem is available in Additional file 1). These vectors can be computed with adapted NMF schemes that can optionally include normalization of *A* and space bound constraints. Specifically, one can apply the multiplicative update algorithmic scheme (as implemented by ‘nnmf’ Matlab function) with or without normalization of *A* or a UPSO-NMF scheme we designed motivated by others [12, 19, 33, 44, 45] so as to offer to the user the option to include space bounds during the calculation of the *S* and *A* vectors. Finally, from each subsystem only the vector of the cell/tissue type-specific proportions is preserved, all of which are subsequently concatenated into one matrix and rescaled with the sum-to-one constraint to form the final proportion matrix *A*.

### Choosing a solver

Deblender provides different algorithms for solving the series of equations describing the deconvolution model. There is option to employ algorithms like ‘active-set’ and ‘trust region reflective’ that solve linear least-squares problems with bounds or linear constraints (implemented with ‘lsqnonneg’ and ‘lsqlin’ functions in Matlab [46, 47]) or use algorithms like ‘interior point convex’ to solve the problem with quadratic objectives and linear constraints (implemented with ‘quadprog’ function in Matlab [48]). Further, Deblender offers the user the opportunity to explore particle swarm optimization (PSO) as an alternative for solving the series of equations. PSO was introduced by Eberhart and Kennedy [49] and is inspired by behavior patterns seen in socially organized colonies to probe the search space so as to find the optimal solution. In PSO the population is called ‘swarm’ and the individuals (search points) are called ‘particles’. Each particle moves in the search space with a velocity adapted iteratively. Also, each particle keeps history of its best position during iterations, i.e., the position with the best objective function value and simultaneously shares this information with the other particles of the swarm. In the global variants of PSO, the neighborhood of each particle is the whole swarm. In the local variants, the neighborhoods are substantially smaller consisting of few particles. In Deblender, we adapted and tailored to the deconvolution problem the Unified Particle Swarm Optimization (UPSO) scheme [50] that combines the exploration and exploitation properties of both the local and global PSO variants. Finally, with regard to Non-negative Matrix Factorization (NMF), we apply the multiplicative update algorithm (‘nnmf’ Matlab function) and also versions of this adapted to use in Deblender and an adapted UPSO-NMF scheme.

## Additional files

Additional file 1: Supplementary results, discussion and methods. (DOCX 2.18 mb)

Additional file 2: Deblender mixture proportions estimates for GSE65135 and TCGA. (XLSX 98 kb)

Additional file 3: Gene Ontology and KEGG pathway enrichment results. (XLSX 690 kb)

## Abbreviations

BRCA: Breast invasive carcinoma
CIBERSORT: Cell-type Identification By Estimating Relative Subsets Of RNA Transcripts
CV: Coefficient of variation
DSA: Digital sorting algorithm
GEO: Gene expression omnibus
GO: Gene ontology
KEGG: Kyoto encyclopedia of genes and genomes
MAS: MicroArray Suite
MDL: Minimum description length
MMAD: Microarray microdissection with analysis of differences
mRMSE: mean Root Mean Squared Error
MSE: Mean squared error
NMF: Non-negative matrix factorization
PSO: Particle swarm optimization
RMA: Robust multichip average
RMSE: Root mean squared error
SE: Standard error
UPSO: Unified particle swarm optimization
