# Supplementary material for: Deblender: a semi−/unsupervised multi-operational computational method for complete deconvolution of expression data from heterogeneous samples
Source: BMC Bioinformatics. 2018 Nov 7;19:408. doi: 10.1186/s12859-018-2442-5 (PMC6223087; doi:10.1186/s12859-018-2442-5)
Supplement: Supplementary file 1 — Supplementary results, discussion and methods. (DOCX 2.18 mb) [file 12859_2018_2442_MOESM1_ESM.docx]

# Results (Supplementary)

## Estimating mixture proportions in benchmark expression data

**GSE11058:** The dataset includes 12 mixed samples composed of the human immune cell lines Jurkat, IM-9, Raji and THP-1 in known proportions. A set of 584 signature probes downloaded by CIBERSORT was used. CIBERSORT was run with the full signature matrix while the complete semi-supervised methods – those that demand putative marker lists – with the probes that were expressed at least 5-fold higher in the respective cell type relative to any of the other cell types. Nevertheless, we also tested the performance of complete semi-supervised methods with all signature probes so as to check how the ‘contamination’ with non-putative markers affects the result (Table S9). Figure S1 shows that the complete semi-supervised mode of Deblender-S1/DSA, NMF-CELLMIX and MMAD performed in line with the partial CIBERSORT. When switching to complete unsupervised mode, Deblender* (for other settings see Tables S4 and S5) outperformed MMAD* (see also Tables S6 and S7) and all semi-supervised and partial methods.

**GSE19380:** The microarray dataset includes 10 mixed samples composed of the rat primary neuronal, astrocytic, oligodendrocytic and microglial cell cultures. Initially, we tested all methods on a subset of 5 samples that were determined to be of good quality. We examined 17 marker probes extracted by Kuhn *et al.* [1] but preserved only the 14 markers that correspond to the first three cell types (i.e., without microglial). It is evident from Figure S2 that the complete semi-supervised mode of Deblender-S1/DSA, NMF-CELLMIX and MMAD as well as Deblender* performed similar to the partial CIBERSORT. When switching to complete unsupervised mode, Deblender* performed better than MMAD* (see also Tables S4, S5, S6, S7). Also, it is worth commenting that when all probes were considered (i.e., both annotated and un-annotated) the performance of Deblender* (default setting – S1) was similarly high ($r = 0.96$, see Table S1). Moreover, we tested both methods with the full mixed dataset of 10 samples to assess performance in a poor quality mixture dataset and Deblender* outperformed MMAD* (see Table S10).

**Deblender and MMAD performance in alternative settings:**

We checked the performance of quadratic programming [2] and Unified Particle Swarm Optimization (UPSO) [3], since Deblender-S1 and DSA provide approximation solutions for the same equation system, and both converged to the same solution for all datasets analyzed.

Deblender* (S1&S2), was run both across all genes of the mixed dataset and on a set of genes closest to the cluster exemplars. The latter case showed improved performance (relative to S1) for all the benchmark datasets (Table S4). Moreover, we tested the performance of Deblender* (S1&S2) without applying the constraints on the cell/tissue type-specific expression matrix $S$ during stage II (S2) on the set that includes only genes belonging to the cluster subsets (Table S14). We observed in the benchmark datasets that the imposed constraints improved the performance of Deblender* in two datasets.

Also, MMAD* was tested on all datasets using both default (1% top gene variant percentile) and with three alternative percentiles (3%, 5% and 7%). For most datasets, the best results were obtained using the 5% and 7% options (Table S6).

MMAD* showed no improvement when tested on the microarray datasets with the ‘customized’ filtering (i.e., removing genes with expression outside the range $[2^{4}-2^{14} ]$ as discussed in other studies [4, 5]) (Table S7).

We explored the effect of technical replicates since it has been suggested that averaging technical replicates in gene expression analysis can lower the effect of measurement error [6]. We tested Deblender* and MMAD* on GSE19830 and GSE11058 by aggregating them prior to any preprocessing. We observed highly similar results for MMAD* before and after aggregation while for Deblender* there was improved performance for GSE11058 (Table S5).

We tested the robustness of Deblender* (S1) by gradually increasing the percentage of Gaussian white noise added to the mixed data (1%, 5%, 10%, 20%, 30%, 50%) and checked the robustness of the produced cluster exemplars during deconvolution. For GSE19830 and RNA-Seq dataset (Table S13), we observed that when we added noise exceeding 20%, the performance dropped. This observation indicates that cluster exemplar profiles preserve up to a certain level of noise the appropriate distinguishable patterns that facilitate the decomposition of the mixed signals.

**
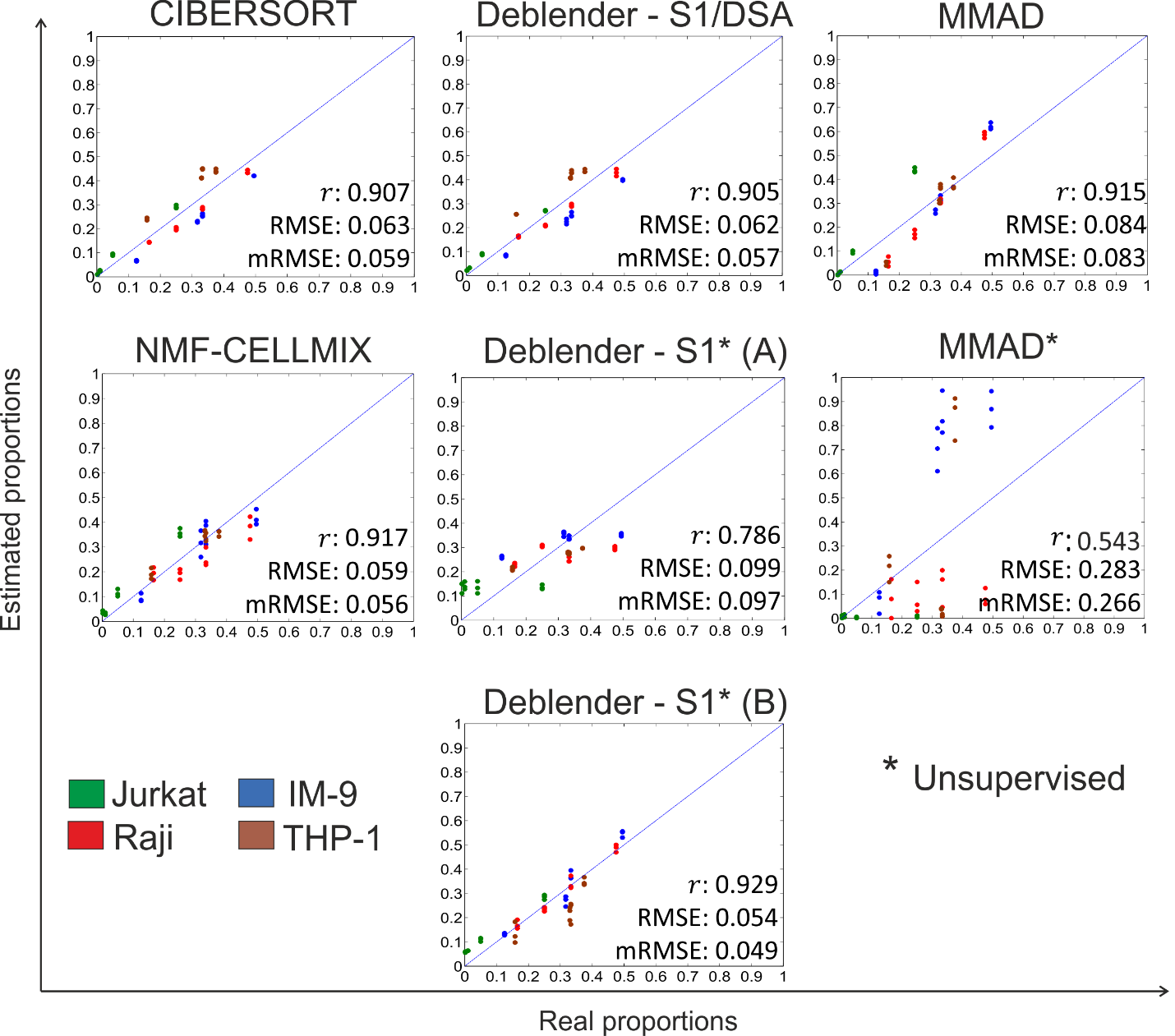
**

**Figure S1.** GSE11058 dataset with 12 mixed samples including 4 immune cell lines (Jurkat, IM-9, Raji, THP-1). Evaluation of methods relative to real mixture proportions based on a set of signature genes (partial: CIBERSORT), on markers extracted from the signature set (complete semi-supervised: Deblender, DSA, MMAD, NMF-CELLMIX) or without prior information (complete unsupervised: Deblender*, MMAD*). Deblender* results are reported with (A) default preprocessing – S1 (B) strict preprocessing – S1.


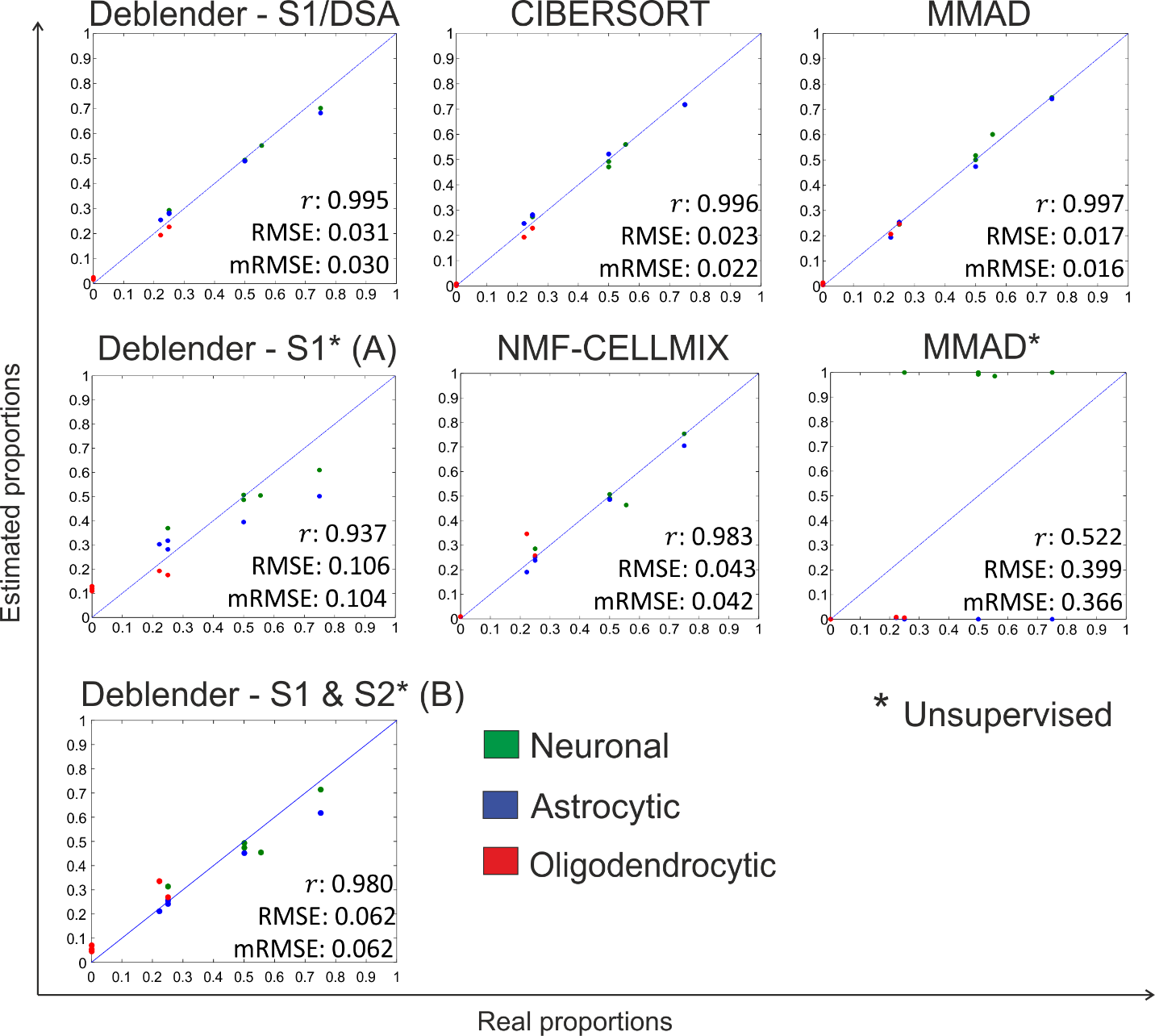


**Figure S2.** GSE19380 dataset with 5 mixed samples including 3 cell cultures (neuronal, astrocytic, oligodendrocytic). Evaluation of methods relative to real mixture proportions based on marker genes (partial: CIBERSORT, complete semi-supervised: Deblender, DSA, MMAD, NMF-CELLMIX) or without a priori information (complete unsupervised: Deblender*, MMAD*). Deblender* results are reported with (A) default preprocessing – S1 (B) with default preprocessing – S1&S2.


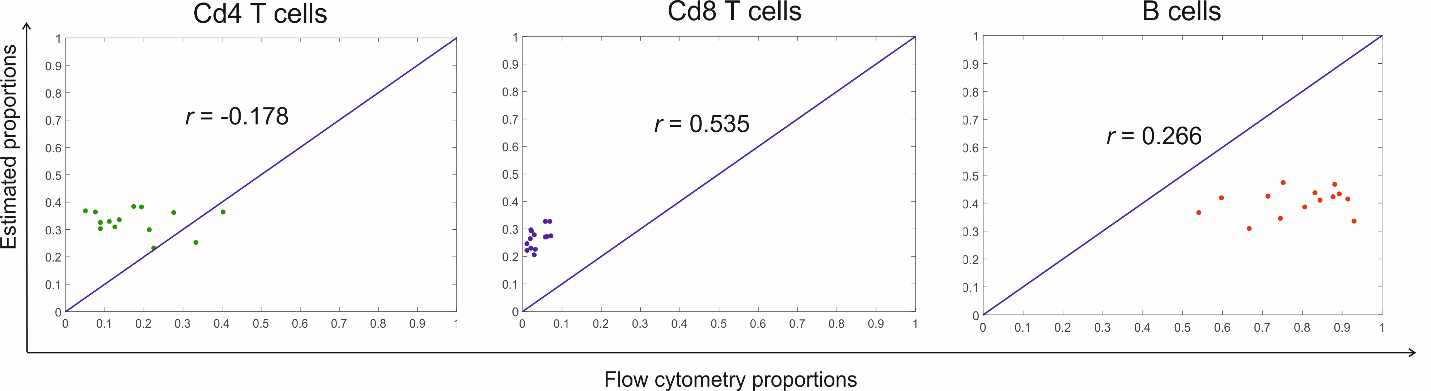


**Figure S3.** Deblender* performance for each cell type separately with regard to GSE65135.


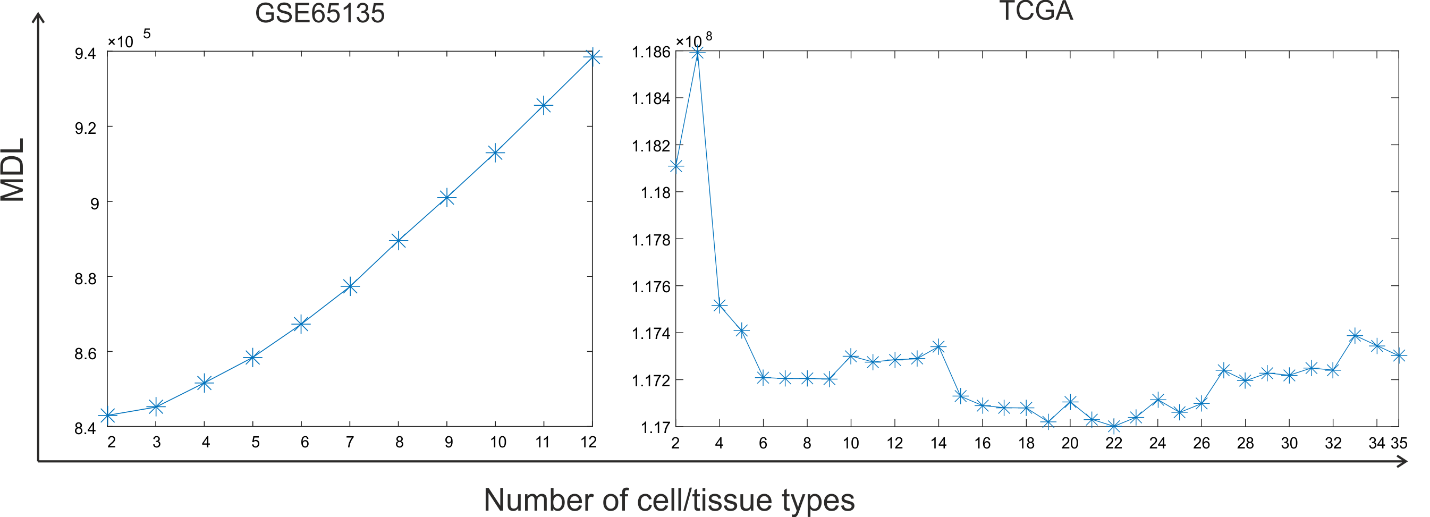


**Figure S4.** MDL estimation for GSE65135 and TCGA ($CV\geq0.4$, customized dataset).


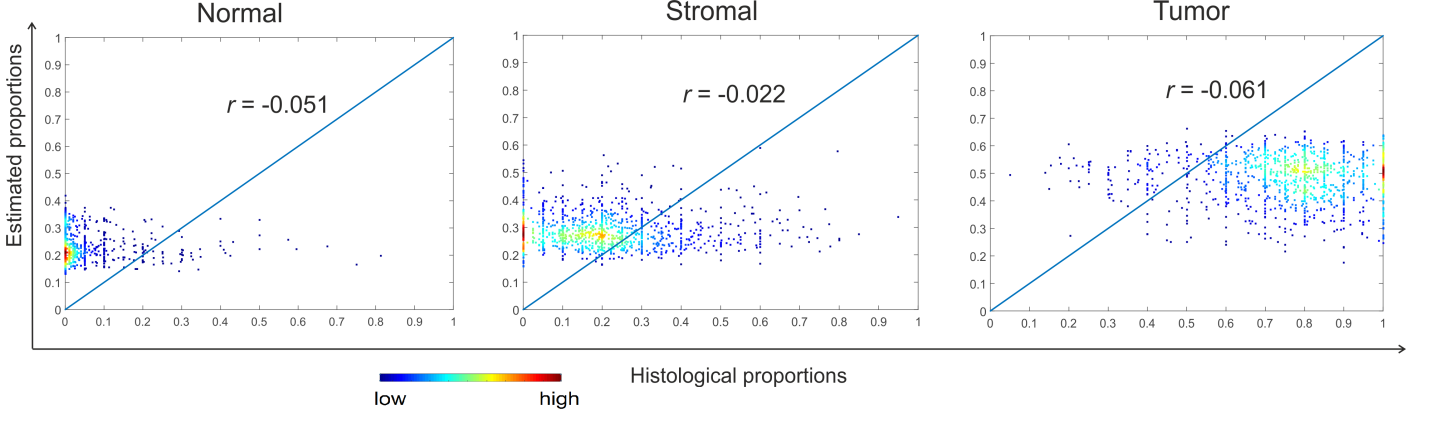


**Figure S5.** Deblender* performance for each tissue component separately with regard to TCGA dataset (density scatterplots are provided).


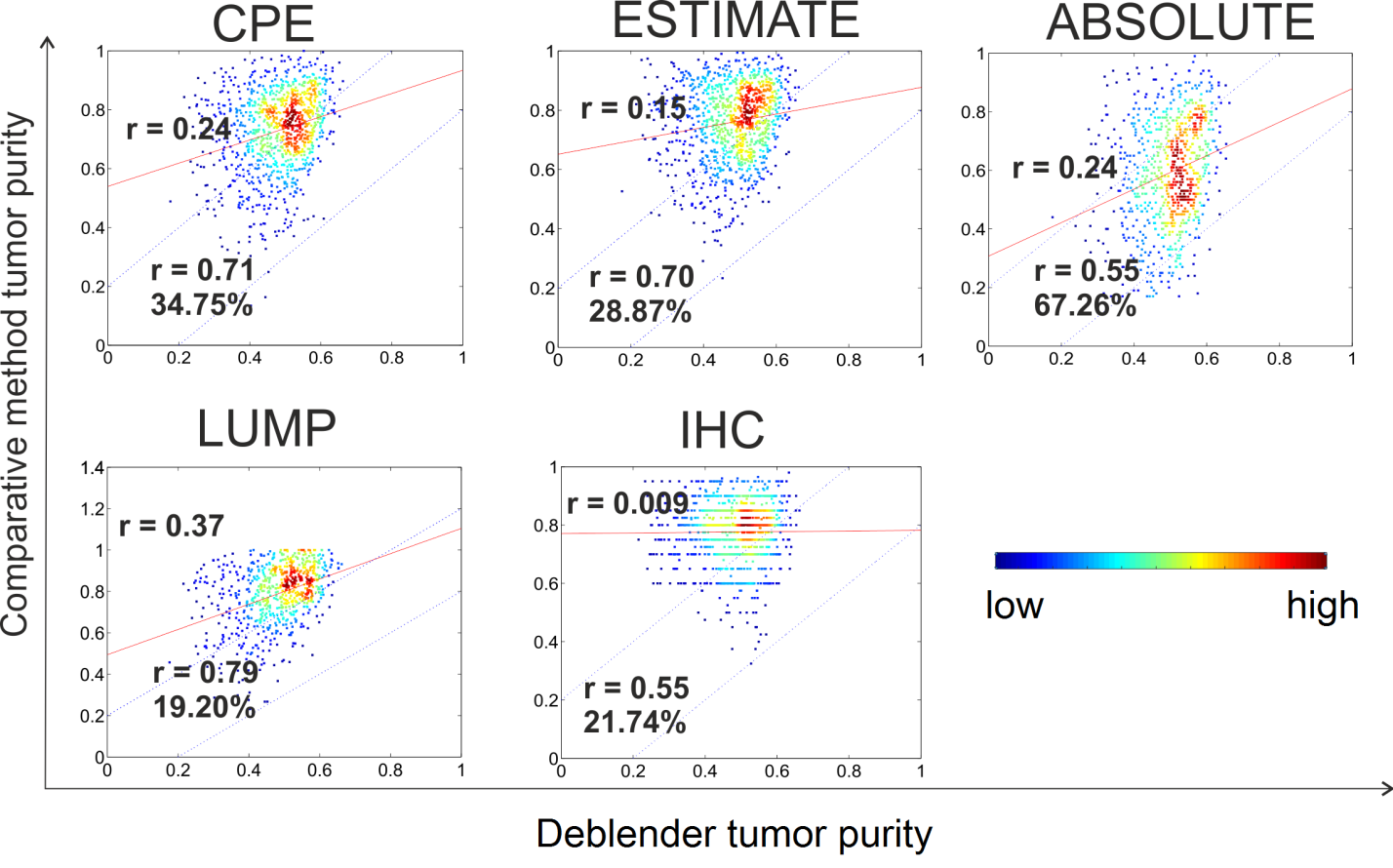


**Figure S6.** Density scatterplots^†^ depicting the tumor purities estimated by Deblender* in the three-tissue-component model (S1, customized dataset) and four other comparative methods with one additional consensus (CPE) for the TCGA breast cancer RNA-Seq samples. The red line represents the linear fit. In the sub-area, where Deblender* estimations deviate $\pm0.2$ from the comparative methods’ estimations, we report the related Pearson correlation and the percentage of samples included.

^†^Density scatterplots were created based on the ‘dscatter.m’ function by Robert Henson (https://www.mathworks.com/matlabcentral/fileexchange/8430-flow-cytometry-data-reader-and-visualization)


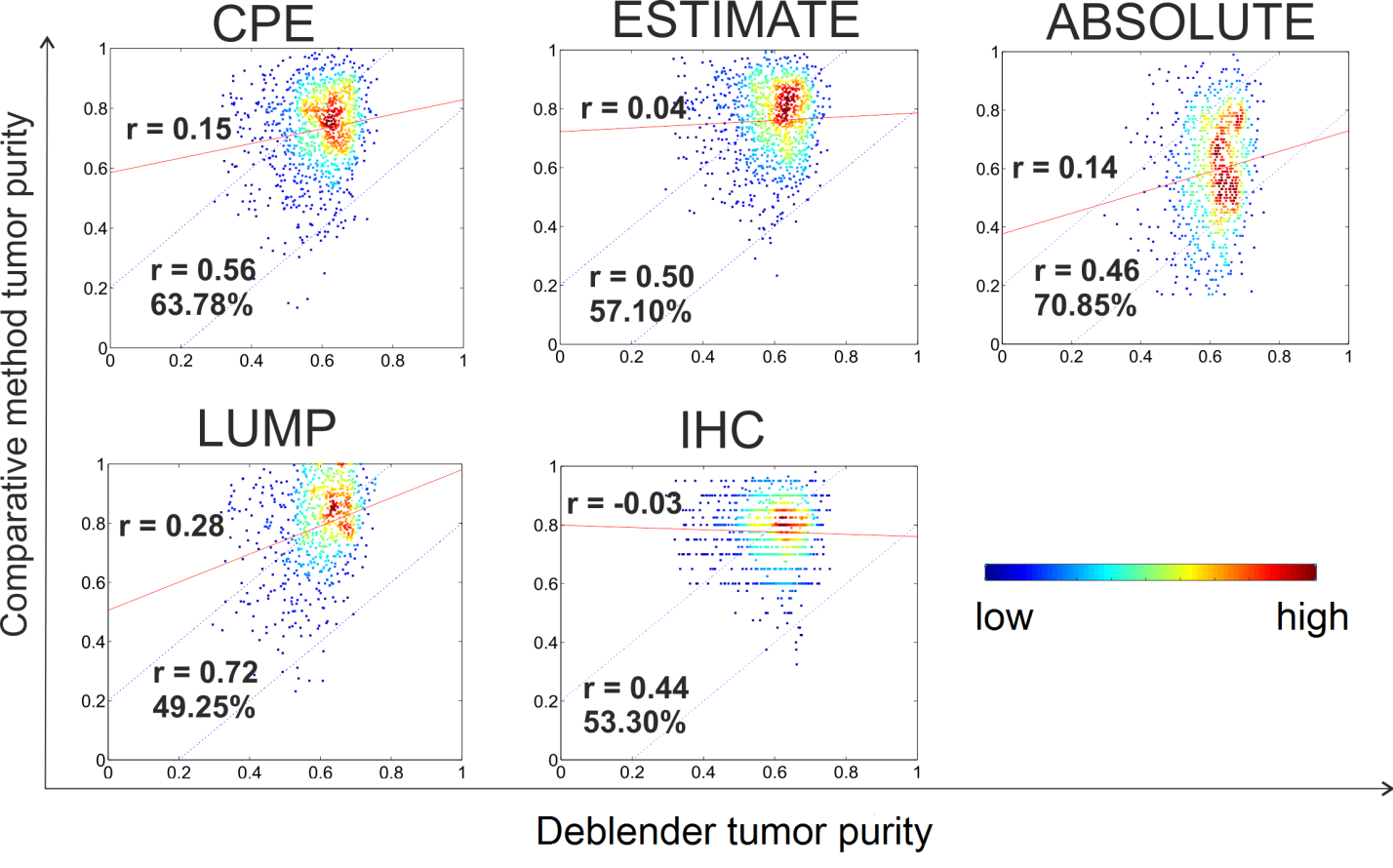


**Figure S7.** Density scatterplots depicting the tumor purities estimated by Deblender* in the two-tissue-component model (S1, customized dataset) and four other comparative methods with one additional consensus (CPE) for the TCGA breast cancer RNA-Seq samples. The red line represents the linear fit. In the sub-area, where Deblender* estimations deviate $\pm0.2$ from the comparative methods’ estimations, we report the related Pearson correlation and the percentage of samples included.


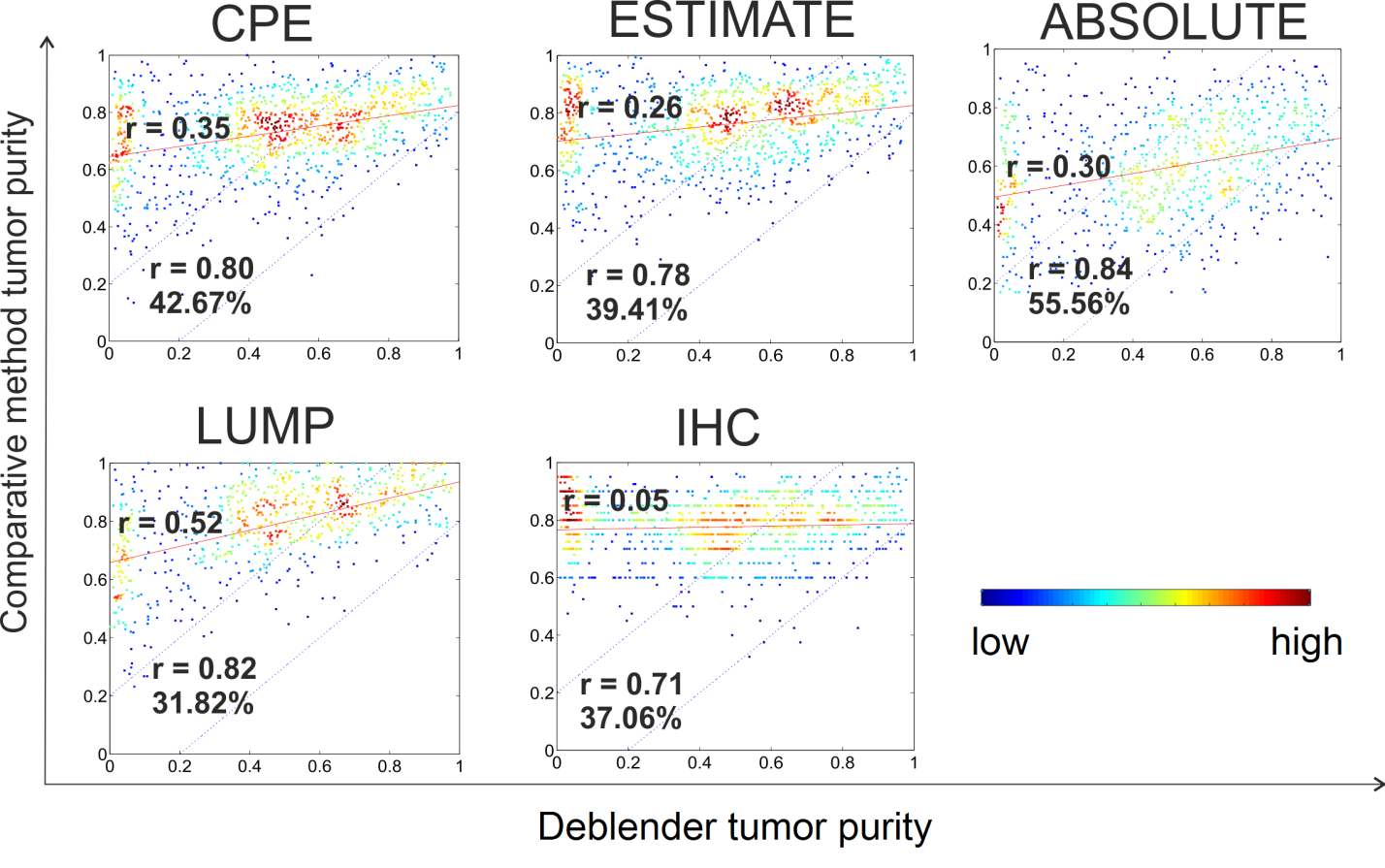


**Figure S8.** Density scatterplots depicting the tumor purities estimated by Deblender* in the two-tissue-component model (S1, $CV\geq3$, customized dataset) and four other comparative methods with one additional consensus (CPE) for the TCGA breast cancer RNA-Seq samples. The red line represents the linear fit. In the sub-area, where Deblender* estimations deviate $\pm0.2$ from the comparative methods’ estimations, we report the related Pearson correlation and the percentage of samples included.


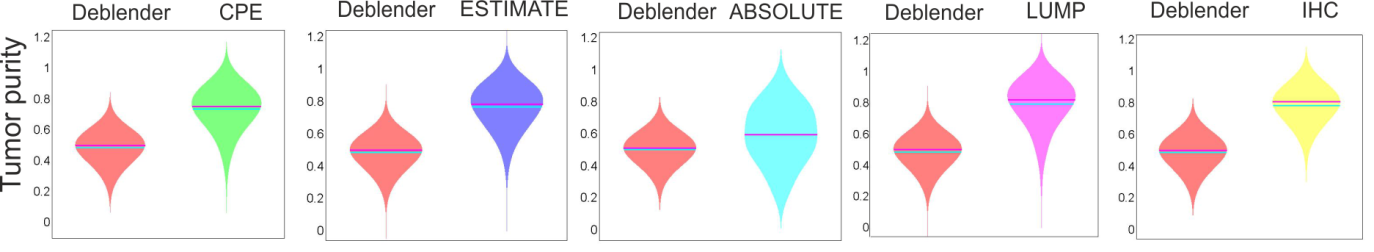


**Figure S9.** Violin plots^†^ showing the distribution of tumor purities estimated by Deblender* in the three-tissue-component model (S1, customized dataset) and four other comparative methods with one additional consensus one (CPE) for the TCGA breast cancer RNA-Seq samples. The number of samples examined in each pairwise comparison is different, therefore Deblender* distribution is adapted accordingly in each case. Cyan: mean, Magenta: median.

^†^Violin plots were made with function ‘violin.m’ by Hoffmann H, 2015: Simple violin plot using matlab default kernel density estimation (https://se.mathworks.com/matlabcentral/fileexchange/45134-violin-plot)


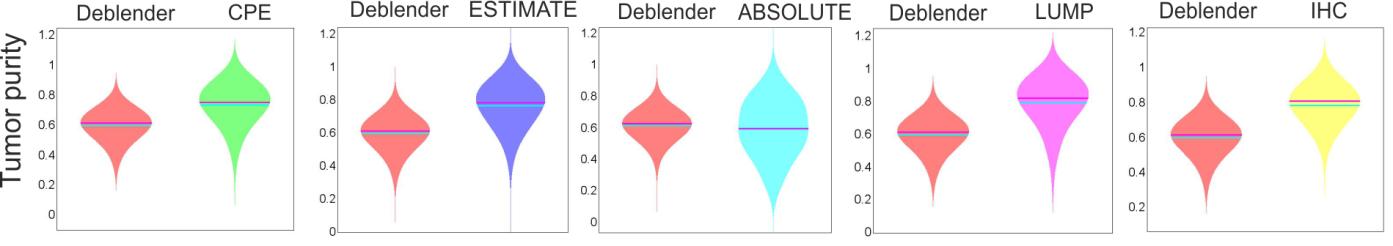


**Figure S10.** Violin plots showing the distribution of tumor purities estimated by Deblender* in the two-tissue-component model (S1, customized dataset) and four other comparative methods with one additional consensus one (CPE) for the TCGA breast cancer RNA-Seq samples. The number of samples examined in each pairwise comparison is different, therefore Deblender* distribution is adapted accordingly in each case. Cyan: mean, Magenta: median


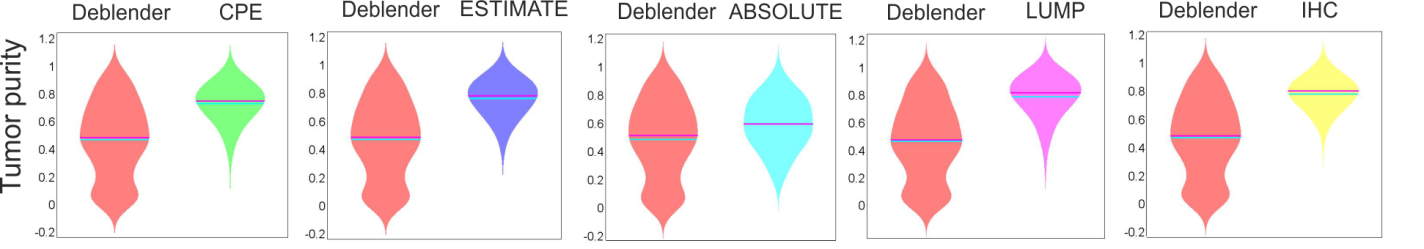
**Figure S11.** Violin plots showing the distribution of tumor purities estimated by Deblender* in the two-tissue-component model (S1, $CV\geq3$, customized dataset) and four other comparative methods with one additional consensus one (CPE) for the TCGA breast cancer RNA-Seq samples. The number of samples examined in each pairwise comparison is different, therefore Deblender* distribution is adapted accordingly in each case. Cyan: mean, Magenta: median.

**
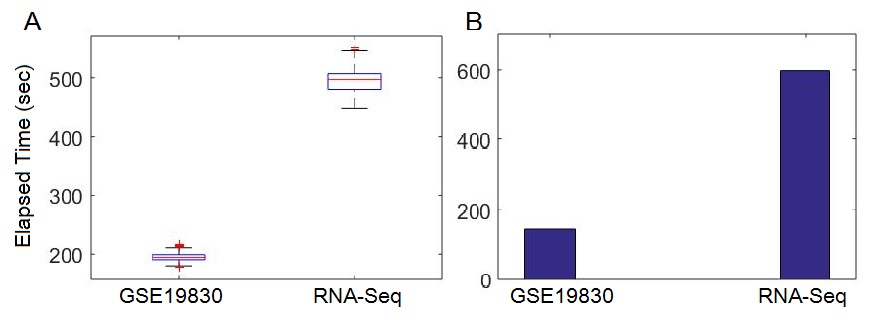
**

**Figure S12.** (a) Elapsed time for calculating proportions (100 runs per dataset) in unsupervised mode (S1&S2) with default settings as described in Methods section. (b) Elapsed time for calculating MDL criterion with $k$ ranging from 2 to 8 and settings as described in Results section.

| **Dataset** | **Deblender*** | | |
| --- | --- | --- | --- |
|  | **Pearson (*r*)** | **RMSE** | **Mean RMSE** |
| GSE19830 | 0.8953 | 0.1459 | 0.1422 |
| GSE11058 | 0.7859 | 0.0994 | 0.0977 |
| GSE19380 | 0.9629 | 0.1196 | 0.1191 |
| RNA-Seq | 0.9095 | 0.1149 | 0.1139 |

**Table S1.** Performance results of the unsupervised mode of Deblender* in benchmark datasets (default setting – S1) without probe processing. In particular, all annotated and un-annotated probes were considered (except control AFFX- probes) and without choosing one probe per gene identifier.

| **Dataset** | **Deblender*** | | |
| --- | --- | --- | --- |
|  | **Pearson (*r*)** | **RMSE** | **Mean RMSE** |
| GSE19830 | 0.7204 | 0.1746 | 0.1685 |
| GSE11058 | 0.7478 | 0.0985 | 0.0958 |
| GSE19380 | 0.8959 | 0.1392 | 0.1374 |
| RNA-Seq | 0.8944 | 0.1125 | 0.1113 |

**Table S2.** Performance results of the unsupervised mode (S1) of Deblender* in preprocessed benchmark datasets without gene filtering.

| **Method** | **Pearson (*r*)** | **RMSE** | **Mean RMSE** |
| --- | --- | --- | --- |
| Deblender - S1(S1&S2) /DSA | 0.9650 (0.9938) | 0.0716 (0.0541) | 0.0689 (0.0538) |
| MMAD | 0.9483 | 0.0702 | 0.0660 |
| NMF-CELLMIX | 0.9917 | 0.0529 | 0.0528 |
| CIBERSORT | 0.9914 | 0.0459 | 0.0453 |

**Table S3.** GSE19830 dataset. Comparing proportion results of partial and complete semi-supervised methods relative to ground truth based on 171 signature probes identified by CIBERSORT, all of which were 5-fold expressed in the respective tissue relative to any of the other tissues.

| **Dataset** | **Deblender* (default – S1&S2)** | | |
| --- | --- | --- | --- |
|  | **Pearson (*r*)** | **RMSE** | **Mean RMSE** |
| GSE19830 | 0.8367 (0.9404) | 0.1436 (0.1163) | 0.1349 (0.1116) |
| GSE11058 | 0.8074 (0.8133) | 0.1027 (0.0948) | 0.0974 (0.0905) |
| GSE19380 | 0.9733 (0.9588) | 0.1135 (0.1316) | 0.1122 (0.1299) |
| RNA-Seq | 0.9613 (0.9197) | 0.0739 (0.0896) | 0.0720 (0.0852) |

**Table S4:** Performance evaluation of Deblender* in the unsupervised mode (default - S1&S2). In S2 the proportion result of S1 was used as initialization for one of the replicated runs and only the mixed data of the subsets of genes closest to cluster exemplars were examined. The results from NMF analysis are reported with normalization of the final estimated factors or without (in parenthesis).

| **Dataset** | **Deblender* – default S1 (S1&S2)** | | | **MMAD* 1% (3%, 5%, 7%)** | | |
| --- | --- | --- | --- | --- | --- | --- |
|  | **Pearson (*r*)** | **RMSE** | **Mean RMSE** | **Pearson (*r*)** | **RMSE** | **Mean RMSE** |
| GSE19830 | 0.8070 (0.9795) | 0.1487 (0.0586) | 0.1456 (0.0563) | 0.6801 (0.6911, 0.8892,  0.8580) | 0.2868 (0.2902, 0.1026, 0.1153) | 0.2557 (0.2570, 0.0975, 0.1060) |
| GSE11058 | 0.9618 | 0.0992 | 0.0939 | 0.5307 (0.6856, 0.7046,  0.7288) | 0.2340 (0.1938, 0.1662, 0.1422) | 0.2128 (0.1824,  0.1533,  0.1374) |

**Table S5.** Performance evaluation of Deblender* (default – S1/S1&S2) and MMAD* (different percentiles for top variable genes) in the unsupervised mode after averaging the profiles of the technical replicates before any further processing (i.e., choosing one probe per gene identifier and deconvolution analysis settings).

| **Dataset** | **3%** | | | **5%** | | | **7%** | | |
| --- | --- | --- | --- | --- | --- | --- | --- | --- | --- |
|  | **Pearson (*r*)** | **RMSE** | **Mean RMSE** | **Pearson (*r*)** | **RMSE** | **Mean RMSE** | **Pearson (*r*)** | **RMSE** | **Mean RMSE** |
| GSE19830 | 0.6903 | 0.2937 | 0.2636 | 0.8801 | 0.1064 | 0.1001 | 0.8485 | 0.1192 | 0.1085 |
| GSE11058 | 0.4869 | 0.2217 | 0.2152 | 0.5192 | 0.2100 | 0.2042 | 0.5578 | 0.2025 | 0.1900 |
| GSE19380 | 0.8912 | 0.1290 | 0.1272 | 0.9082 | 0.1131 | 0.1104 | 0.9663 | 0.0753 | 0.0751 |
| RNA-Seq | 0.2962 | 0.2901 | 0.2639 | 0.3422 | 0.2703 | 0.2525 | 0.4122 | 0.2497 | 0.2256 |
| RNA-Seq (under-determined) | 0.5926 | 0.2583 | 0.2400 | 0.5318 | 0.2782 | 0.2402 | 0.5684 | 0.2763 | 0.2306 |
| GSE65135 | 0.6458 | 0.2816 | 0.2800 | 0.7249 | 0.2463 | 0.2457 | 0.7160 | 0.2474 | 0.2468 |
| TCGA | 0.5140 | 0.3484 | 0.3349 | 0.5676 | 0.3398 | 0.3077 | 0.5651 | 0.3444 | 0.3185 |

**Table S6.** MMAD* (unsupervised mode) mixture proportion estimation results with different top variant gene percentiles other than the default 1%.

| **Dataset** | **1%** | | | **3%** | | | **5%** | | | **7%** | | |
| --- | --- | --- | --- | --- | --- | --- | --- | --- | --- | --- | --- | --- |
|  | **Pearson (*r*)** | **RMSE** | **Mean RMSE** | **Pearson (*r*)** | **RMSE** | **Mean RMSE** | **Pearson (*r*)** | **RMSE** | **Mean RMSE** | **Pearson (*r*)** | **RMSE** | **Mean RMSE** |
| GSE19830 | 0.6795 | 0.2841 | 0.2521 | 0.6932 | 0.2912 | 0.2600 | 0.8711 | 0.1105 | 0.1049 | 0.8651 | 0.1122 | 0.1036 |
| GSE11058 | 0.5041 | 0.3183 | 0.2984 | 0.7359 | 0.1571 | 0.1495 | 0.7200 | 0.1563 | 0.1419 | 0.7406 | 0.1587 | 0.1493 |
| GSE19380 | 0.5090 | 0.3572 | 0.3224 | 0.8727 | 0.1393 | 0.1375 | 0.9256 | 0.0998 | 0.0974 | 0.9611 | 0.0781 | 0.0780 |
| GSE65135 | 0.1397 | 0.3784 | 0.3662 | 0.2180 | 0.3558 | 0.3451 | 0.1440 | 0.3615 | 0.3499 | 0.1325 | 0.3638 | 0.3508 |
| TCGA* | 0.3019 | 0.3899 | 0.3809 | 0.4188 | 0.3535 | 0.3449 | 0.5600 | 0.3401 | 0.3133 | 0.5842 | 0.3400 | 0.3044 |

**Table S7.** MMAD* (unsupervised mode) mixture proportion estimation results with different top variant gene percentiles after preserving only the genes of the microarray datasets that fall into the expression range $\left[ 2^{4}-2^{14} \right]$. * for TCGA RNA-Seq data a ‘customized’ filtering was applied (see ‘Methods’).

| **GSE19830** | **Real** | | | **Estimated (S1&S2)** | | |
| --- | --- | --- | --- | --- | --- | --- |
|  | **Liver** | **Brain** | **Lung** | **Liver** | **Brain** | **Lung** |
| GSM495218 | 0.0500 | 0.2500 | 0.7000 | 0.0007 | 0.3372 | 0.6620 |
| GSM495219 | 0.0500 | 0.2500 | 0.7000 | 0.0019 | 0.3410 | 0.6571 |
| GSM495220 | 0.0500 | 0.2500 | 0.7000 | 0.0023 | 0.3399 | 0.6578 |
| GSM495221 | 0.7000 | 0.0500 | 0.2500 | 0.6104 | 0.0957 | 0.2939 |
| GSM495222 | 0.7000 | 0.0500 | 0.2500 | 0.6049 | 0.0977 | 0.2974 |
| GSM495223 | 0.7000 | 0.0500 | 0.2500 | 0.6125 | 0.0915 | 0.2961 |
| GSM495224 | 0.2500 | 0.7000 | 0.0500 | 0.2016 | 0.6885 | 0.1099 |
| GSM495225 | 0.2500 | 0.7000 | 0.0500 | 0.1971 | 0.6897 | 0.1132 |
| GSM495226 | 0.2500 | 0.7000 | 0.0500 | 0.2059 | 0.6840 | 0.1101 |
| GSM495227 | 0.7000 | 0.2500 | 0.0500 | 0.5735 | 0.3077 | 0.1187 |
| GSM495228 | 0.7000 | 0.2500 | 0.0500 | 0.5742 | 0.3061 | 0.1198 |
| GSM495229 | 0.7000 | 0.2500 | 0.0500 | 0.5733 | 0.3062 | 0.1204 |
| GSM495230 | 0.4500 | 0.4500 | 0.1000 | 0.4005 | 0.4522 | 0.1473 |
| GSM495231 | 0.4500 | 0.4500 | 0.1000 | 0.3949 | 0.4562 | 0.1489 |
| GSM495232 | 0.4500 | 0.4500 | 0.1000 | 0.3977 | 0.4551 | 0.1472 |
| GSM495233 | 0.5500 | 0.2000 | 0.2500 | 0.4909 | 0.2387 | 0.2704 |
| GSM495234 | 0.5500 | 0.2000 | 0.2500 | 0.4918 | 0.2411 | 0.2671 |
| GSM495235 | 0.5500 | 0.2000 | 0.2500 | 0.4856 | 0.2459 | 0.2685 |
| GSM495236 | 0.5000 | 0.3000 | 0.2000 | 0.4455 | 0.3252 | 0.2293 |
| GSM495237 | 0.5000 | 0.3000 | 0.2000 | 0.4458 | 0.3250 | 0.2292 |
| GSM495238 | 0.5000 | 0.3000 | 0.2000 | 0.4455 | 0.3242 | 0.2303 |
| GSM495239 | 0.5500 | 0.3000 | 0.1500 | 0.4768 | 0.3295 | 0.1937 |
| GSM495240 | 0.5500 | 0.3000 | 0.1500 | 0.4750 | 0.3324 | 0.1926 |
| GSM495241 | 0.5500 | 0.3000 | 0.1500 | 0.4752 | 0.3305 | 0.1942 |
| GSM495242 | 0.5000 | 0.4000 | 0.1000 | 0.4339 | 0.4181 | 0.1480 |
| GSM495243 | 0.5000 | 0.4000 | 0.1000 | 0.4357 | 0.4159 | 0.1484 |
| GSM495244 | 0.5000 | 0.4000 | 0.1000 | 0.4337 | 0.4159 | 0.1504 |
| GSM495245 | 0.6000 | 0.3500 | 0.0500 | 0.5057 | 0.3918 | 0.1025 |
| GSM495246 | 0.6000 | 0.3500 | 0.0500 | 0.5087 | 0.3836 | 0.1077 |
| GSM495247 | 0.6000 | 0.3500 | 0.0500 | 0.5045 | 0.3905 | 0.1050 |
| GSM495248 | 0.6500 | 0.3400 | 0.0100 | 0.5458 | 0.3971 | 0.0570 |
| GSM495249 | 0.6500 | 0.3400 | 0.0100 | 0.5465 | 0.3987 | 0.0548 |
| GSM495250 | 0.6500 | 0.3400 | 0.0100 | 0.5443 | 0.3990 | 0.0567 |

**Table S8.** Side-by-side report of Deblender* estimates (unsupervised mode, S1&S2) versus real proportions in GSE19830 dataset (Table related to Figure 2).

| **Method** | **Pearson (*r*)** | **RMSE** | **Mean RMSE** |
| --- | --- | --- | --- |
| Deblender – S1/DSA | 0.9120 | 0.0632 | 0.0602 |
| MMAD | 0.9183 | 0.0827 | 0.0813 |
| NMF-CELLMIX | 0.9174 | 0.0643 | 0.0613 |

**Table S9.** GSE11058 dataset. Comparing proportion results of complete semi-supervised methods relative to ground truth based on 584 signature probes downloaded by CIBERSORT.

| **Method** | **Pearson correlation (*r*)** | **RMSE** | **Mean RMSE** |
| --- | --- | --- | --- |
| Deblender* (default-S1) | 0.7208 | 0.1704 | 0.1627 |
| MMAD* 1% (3%, 5%, 7%) | 0.3194 (0.3174, 0.3170, 0.3189) | 0.3865 (0.3824, 0.3767, 0.3741) | 0.3648 (0.3647, 0.3620, 0.3609) |

**Table S10.** GSE19380 dataset. Comparing proportion results (for three cell types) of Deblender* and MMAD* (unsupervised mode) on the complete mixed expression dataset (10 samples).

| **RNA-Seq** | **Real** | | | | | **Estimated (S1)** | | | | |
| --- | --- | --- | --- | --- | --- | --- | --- | --- | --- | --- |
|  | **Brain** | **Muscle** | **Lung** | **Liver** | **Heart** | **Brain** | **Muscle** | **Lung** | **Liver** | **Heart** |
| Sample 1 | 0.0463 | 0.0323 | 0.0805 | 0.0747 | 0.7662 | 0.0872 | 0.1056 | 0.1573 | 0.1342 | 0.5158 |
| Sample 2 | 0.0606 | 0.1156 | 0.0278 | 0.6960 | 0.1000 | 0.0850 | 0.1302 | 0.1135 | 0.4898 | 0.1815 |
| Sample 3 | 0.0728 | 0.6058 | 0.1051 | 0.1262 | 0.0900 | 0.0983 | 0.3507 | 0.1443 | 0.1594 | 0.2474 |
| Sample 4 | 0.0709 | 0.0887 | 0.7242 | 0.0975 | 0.0188 | 0.1126 | 0.1305 | 0.4661 | 0.1694 | 0.1214 |
| Sample 5 | 0.6672 | 0.1347 | 0.0486 | 0.0674 | 0.0821 | 0.3712 | 0.1675 | 0.1230 | 0.1307 | 0.2077 |
| Sample 6 | 0.1368 | 0.2181 | 0.1764 | 0.3678 | 0.1010 | 0.1270 | 0.1825 | 0.1864 | 0.3056 | 0.1985 |
| Sample 7 | 0.0780 | 0.2100 | 0.2800 | 0.1603 | 0.2717 | 0.1047 | 0.1820 | 0.2432 | 0.1888 | 0.2814 |
| Sample 8 | 0.1250 | 0.3997 | 0.1830 | 0.1198 | 0.1726 | 0.1245 | 0.2650 | 0.1896 | 0.1608 | 0.2602 |
| Sample 9 | 0.2309 | 0.1230 | 0.5723 | 0.0214 | 0.0524 | 0.1858 | 0.1518 | 0.3882 | 0.1199 | 0.1543 |
| Sample 10 | 0.4284 | 0.3242 | 0.0913 | 0.0644 | 0.0917 | 0.2637 | 0.2423 | 0.1419 | 0.1267 | 0.2254 |

**Table S11.** Side-by-side report of Deblender* estimates (unsupervised mode, S1) versus real proportions in RNA-Seq dataset (Table related to Figure 3).

| **Method** | **RNA-Seq** | | |
| --- | --- | --- | --- |
|  | **Pearson (*r*)** | **RMSE** | **mRMSE** |
| CIBERSORT | 0.8979 | 0.0906 | 0.0828 |
| DECONRNASeq | 0.9753 | 0.0444 | 0.0438 |
| Deblender-S1/DSA | 0.8567 | 0.1062 | 0.1003 |
| NMF-CELLMIX | 0.3846 | 0.2164 | 0.1893 |
| MMAD | 0.9895 | 0.0371 | 0.0365 |
| Deblender* (default-S1) | 0.9711 | 0.1099 | 0.1093 |
| MMAD* 1 % (3%, 5%, 7%) | 0.7375 (0.9967, 0.9947, 0.9943) | 0.1711 (0.0239, 0.0316, 0.0217) | 0.1335 (0.0235, 0.0308, 0.0191) |

**Table S12.** Performance results of the partial and complete semi-/unsupervised methods for the RNA-Seq dataset after adding an offset of 1 prior to analysis.

| **Noise %** | **GSE19830** | | | **RNA-Seq** | | |
| --- | --- | --- | --- | --- | --- | --- |
|  | **Pearson (*r*)** | **RMSE** | **mRMSE** | **Pearson (*r*)** | **RMSE** | **mRMSE** |
| 1 | 0.81 | 0.14 | 0.14 | 0.94 | 0.10 | 0.10 |
| 5 | 0.80 | 0.15 | 0.14 | 0.94 | 0.10 | 0.10 |
| 10 | 0.79 | 0.15 | 0.15 | 0.93 | 0.11 | 0.11 |
| 20 | 0.81 | 0.15 | 0.15 | 0.84 | 0.12 | 0.12 |
| 30 | 0.64 | 0.18 | 0.18 | 0.21 | 0.19 | 0.19 |
| 50 | 0.58 | 0.18 | 0.18 | 0.12 | 0.19 | 0.19 |

**Table S13.** Performance evaluation of the unsupervised mode of Deblender* (default setting – S1) after adding Gaussian white noise to the mixed data in different percentages.

| **Dataset** | **Deblender* (default – S1&S2)** | | |
| --- | --- | --- | --- |
|  | **Pearson (*r*)** | **RMSE** | **Mean RMSE** |
| GSE19830 | 0.8787 | 0.1058 | 0.1023 |
| GSE11058 | 0.5210 | 0.1685 | 0.1614 |
| GSE19380 | 0.8340 | 0.1445 | 0.1394 |
| RNA-Seq | 0.9798 | 0.0611 | 0.0576 |

**Table S14.** Performance evaluation of Deblender* in the unsupervised mode (default - S1&S2). In S2 the proportion result of S1 was used as initialization for one of the replicated runs, no constraints were applied in *S* matrix during iterations and only the mixed data of the subsets of genes closest to cluster exemplars were examined.

# Discussion (Supplementary)

Partial and complete deconvolution methods tested against Deblender: (a) Digital Sorting Algorithm (DSA) is a complete semi-supervised deconvolution method which requires marker gene lists and computes both the mixture proportions and the cell/tissue type-specific expression profiles using quadratic programming [2], (b) Microarray Microdissection with Analysis of Differences (MMAD) is a partial/complete semi-/unsupervised deconvolution method that offers several deconvolution and differential expression pipelines and uses marker gene lists or highly variable genes identified from the mixed dataset [7], (c) the Non-negative Matrix Factorization (NMF) based algorithm by Gaujoux and Seoighe [8,9], part of Cellmix R package, is a complete semi-supervised deconvolution method that modifies common NMF algorithms to estimate mixture proportions and cell/tissue type-specific expression profiles by imposing the expected expression values of known marker genes, (d) DeconRNASeq is a partial deconvolution method, designed for RNA-seq transcriptome data (works also for microarray), which calculates mixture proportions with quadratic programming [10], and (e) Cell-type Identification By Estimating Relative Subsets Of RNA Transcripts (CIBERSORT) is a partial deconvolution approach which defines a gene expression signature matrix based on reference cell/tissue type-specific expression profiles and calculates mixture proportions using a linear support vector regression approach while providing also an empirical P value after testing the null hypothesis that no cell types in the signature matrix are present in the mixture [11].

# Methods (Supplementary)

## Estimating mixture proportions in over-determined cases, Stage II

### Pseudocode (semi-supervised)

1. Input: $X$(gene mixed data), $k$ marker gene sets,$A$ (from Deblender stage I), $S$ (optionally from Deblender stage I), number of iterations ($num\_iter$),number of replicates($repl$)
2. Use multiplicative update algorithm (as in ‘nnmf’ Matlab function)

For i=1:$repl$

Initialize randomly $S_{0}$ (or set $S_{0}=S$ in first replicate), add zeros in the non-cell-type-specific columns of each marker gene set and set $A_{0}$ = $A$ (in first replicate)

While $not convergent$or $iter\leq num\_iter$

Calculate $A$

Calculate $S$

Estimate $D$*

Check for convergence

Set $A_{0}=A, S_{0}=S$

End

End

Retain $A$, $S$ with lowest $D$ across replicates

Normalize$A$, $S$ (optional)

1. Rescale the final $A$ proportion matrix with the sum-to-one constraint for each sample

* D: root-mean-squared residual D between $X$ and $S$*$A$

### Pseudocode (unsupervised)

1. Input: $X$(gene mixed data), number of cell types ($k$),$A$ (from Deblender stage I), $S$ (optionally from Deblender stage I), number of iterations ($num\_iter$), number of replicates ($repl$)
2. Cluster gene profiles in $k$cluster sets, define cluster subsets by choosing for each cluster set the $n$% of genes closest to cluster centroids/medoids
3. Use multiplicative update algorithm (as in ‘nnmf’ Matlab function)

For i=1:$repl$

Initialize randomly $S_{0}$ (or set $S_{0}=S$ in first replicate), add zeros in the non-cell-type-specific columns of each gene cluster subset and set $A_{0}$ = $A$ (in first replicate)

While $not convergent$or $iter\leq num\_iter$

Calculate $A$

Calculate $S$

Estimate $D$*

Check for convergence

Set $A_{0}=A, S_{0}=S$

End

End

Retain $A$, $S$ with lowest $D$ across replicates

Normalize$A$, $S$ (optional)

1. Rescale the final $A$ proportion matrix with the sum-to-one constraint for each sample

* D: root-mean-squared residual D between $X$ and $S$*$A$

## Estimating mixture proportions in under-determined cases

### Pseudocode (semi-supervised)

1. Input: $X_{m}$(marker gene mixed data), number of cell types (k), number of iterations ($num\_iter$), number of replicates ($repl$)
2. For marker set $X_{mi}$corresponding to cell type $i$ (subsystem $i$)

For j=1:$repl$

Initialize $A_{0}, S_{0}$

While $not convergent$or $iter\leq num\_iter$

Calculate vector $A_{i}$(within space bounds in UPSO-NMF)

Calculate vector $S_{mi}$ (within space bounds in UPSO-NMF)

Estimate $D$*

Check for convergence

Set $A_{0}=A_{i}, S_{0}=S_{mi}$

End

End

Retain $A_{i}$, $S_{mi}$ with lowest $D$ across replicates

Normalize$A_{i}$*,* $S_{mi}$ (optional)

End

1. Combine the relevant proportion vectors $A_{i}$ from all subsystems and rescale with sum-to-one constraint for each sample to form the final $A$ proportion matrix

* D: root-mean-squared residual D between $X_{mi}$ and $S_{mi}{*A}_{i}$

### Pseudocode (unsupervised)

1. Input: $X$(gene mixed data), number of cell types (k), number of iterations ($num\_iter$), number of replicates ($repl$)
2. Cluster $X$ into $k$ cluster sets (i.e. $k$ cell types)

Define $k$ cluster subsets after choosing for each cluster set the $n$% of genes closest to cluster centroids/medoids

1. For each cluster subset$X_{ci}$ corresponding to cell type $i$ (subsystem $i$)

For j=1:$repl$

Initialize $A_{0}, S_{0}$

While $not convergent$or $iter\leq num\_iter$

Calculate vector $A_{i}$ (within space bounds in UPSO-NMF)

Calculate vector $S_{ci}$ (within space bounds in UPSO-NMF)

Estimate $D$*

Check for convergence

Set $A_{0}=A_{i}, S_{0}=S_{ci}$

End

End

Retain $A_{i}$, $S_{ci}$ with lowest $D$ across replicates

Normalize$A_{i}$*,* $S_{ci}$(optional)

End

1. Combine the relevant proportion vectors $A_{i}$ from all subsystems and rescale with sum-to-one constraint for each sample to form the final $A$ proportion matrix

* D: root-mean-squared residual D between $X_{ci}$ and $S_{ci}{*A}_{i}$

### Example for estimating proportions in an under-determined deconvolution problem:

In this example, the dataset consists of 4 mixed samples with 5 cell types participating in the mixture. Suppose we have 4 marker genes for the first cell type, 3 markers for the second cell type, 3 markers for the third cell type, 4 markers for the fourth cell type and 3 markers for the fifth cell type. The markers can be either known a priori or can be detected in an unsupervised manner based on clustering. In this case, equation 2 (see manuscript) describing the equations referring to marker genes $X_{m}$ becomes:

$$\left(\begin{matrix} X_{1,1} X_{1,2} X_{1,3} X_{1,4} \\ X_{2,1} X_{2,2} X_{2,3} X_{2,4} \\ X_{3,1} X_{3,2} X_{3,3} X_{3,4} \\ X_{4,1} X_{4,2} X_{4,3} X_{4,4} \\ X_{5,1} X_{5,2} X_{5,3} X_{5,4} \\ X_{6,1} X _{6,2} X_{6,3} X_{6,4} \\ X_{7,1} X_{7,2} X_{7,3} X_{7,4} \\ X_{8,1} X_{8,2} X_{8,3} X_{8,4} \\ X_{9,1} X_{9,2} X_{9,3} X_{9,4} \\ X_{10,1} X_{10,2} X_{10,3} X_{10,4} \\ X_{11,1} X_{11,2} X_{11,3} X_{11,4} \\ X_{12,1} X_{12,2} X_{12,3} X_{12,4} \\ X_{13,1} X_{13,2} X_{13,3} X_{13,4} \\ X_{14,1} X_{14,2} X_{14,3} X_{14,4} \\ X_{15,1} X_{15,2} X_{15,3} X_{15,4} \\ X_{16,1} X_{16,2} X_{16,3} X_{16,4} \\ X_{17,1} X_{17,2} X_{17,3} X_{17,4} \end{matrix}\right)= \left(\begin{matrix} S_{1,1} 0 0 0 0 \\ S_{2,1} 0 0 0 0 \\ S_{3,1} 0 0 0 0 \\ S_{4,1} 0 0 0 0 \\ 0 S_{5,2} 0 0 0 \\ 0 S_{6,2} 0 0 0 \\ 0 S_{7,2} 0 0 0 \\ 0 0 S_{8,3} 0 0 \\ 0 0 S_{9,3} 0 0 \\ 0 0 S_{10,3} 0 0 \\ 0 0 0 S_{11,4} 0 \\ 0 0 0 S_{12,4} 0 \\ 0 0 0 S_{13,4} 0 \\ 0 0 0 S_{14,4} 0 \\ 0 0 0 0 S_{15,5} \\ 0 0 0 0 S_{16,5} \\ 0 0 0 0 S_{17,5} \end{matrix}\right)\bullet\left(\begin{matrix} A_{1,1} A_{1,2} A_{1,3}A_{1,4} \\ A_{2,1} A_{2,2} A_{2,3}A_{2,4} \\ A_{3,1} A_{3,2} A_{3,3}A_{3,4} \\ A_{4,1} A_{4,2} A_{4,3}A_{4,4} \\ A_{5,1} A_{5,2} A_{5,3}A_{5,4} \end{matrix}\right)$$

For example, $X_{1,2}$ refers to the mixed values of the first marker gene in the second sample, $S_{2,1}$ is the pure expression value of the second marker gene in the first cell type and $A_{1,2}$ is the proportion of the first cell type in the second sample. This equation system is further split into 5 sub-systems, each one corresponding to the mixed expression data of the marker genes that refer to a specific cell type. Each subsystem is solved separately. Let us focus on the first subsystem:

$$\left(\begin{matrix} X_{1,1} X_{1,2} X_{1,3} X_{1,4} \\ X_{2,1} X_{2,2} X_{2,3} X_{2,4} \\ X_{3,1} X_{3,2} X_{3,3} X_{3,4} \\ X_{4,1} X_{4,2} X_{4,3} X_{4,4} \end{matrix}\right)= \left(\begin{matrix} S_{1,1} 0 0 0 0 \\ S_{2,1} 0 0 0 0 \\ S_{3,1} 0 0 0 0 \\ S_{4,1} 0 0 0 0 \end{matrix}\right)\bullet\left(\begin{matrix} A_{1,1} A_{1,2} A_{1,3}A_{1,4} \\ A_{2,1} A_{2,2} A_{2,3}A_{2,4} \\ A_{3,1} A_{3,2} A_{3,3}A_{3,4} \\ A_{4,1} A_{4,2} A_{4,3}A_{4,4} \\ A_{5,1} A_{5,2} A_{5,3}A_{5,4} \end{matrix}\right)$$

Due to the zero columns of the $S$ matrix, we can omit the estimation of the non-cell-type-specific $A$ values (which do not affect the objective function value during the approximation *S*A*) and the subsystem can be further simplified:

$$\left(\begin{matrix} X_{1,1} X_{1,2} X_{1,3} X_{1,4} \\ X_{2,1} X_{2,2} X_{2,3} X_{2,4} \\ X_{3,1} X_{3,2} X_{3,3} X_{3,4} \\ X_{4,1} X_{4,2} X_{4,3} X_{4,4} \end{matrix}\right)= \left(\begin{matrix} S_{1,1} \\ S_{2,1} \\ S_{3,1} \\ S_{4,1} \end{matrix}\right)\bullet\left(A_{1,1} A_{1,2} A_{1,3}A_{1,4}\right)$$

A solution can be approximated for this subsystem and $S$ and $A$ vectors are computed in order to minimize the objective function that is defined as the root mean squared residual between $X$ and the approximation $S*A$ (used in Matlab ‘nnmf’ function). An approximation solution for this subsystem can be estimated with the adapted NMF schemes. When all proportion vectors from all subsystems are computed, they are concatenated into a single matrix which is further rescaled with the sum-to-one constraint for each sample.

## Benchmark expression datasets

GSE19830: The microarray dataset includes samples of pure rat brain, liver and lung tissue and mixtures of them in 11 different proportions [12]. For each proportion there are 3 technical replicates with 42 total samples (9 tissue-specific and 33 mixed tissue samples). We downloaded from Gene Expression Omnibus (GEO) [13] the available Robust Multichip Average (RMA) normalized data and unlogged them. With regard to partial and complete semi-supervised methods, a set of 237 marker probes was downloaded [2] and a 171 probe signature set was examined, after implementing CIBERSORT’s signature generation process [11] on all annotated probe tissue-specific expression data (based on the rat2302.db annotation R/Bioconductor package [14] after filtering all Affymetrix control probesets) without preprocessing cases with multiple probes per Entrez Id. Based on the average of replicated samples of the tissue-specific expression data, we defined markers two-way: (a) we assigned each probe to a putative tissue based on which tissue each probe displayed the maximal expression value (b) we assigned each probe to a putative tissue after displaying 5-fold expression over any other tissue (as proposed by Newman *et al.* [11]). In this dataset markers from (a) and (b) matched. The preprocessed dataset includes all the probes that could be mapped to an Entrez identifier and in case of multiple probes per Entrez ID the probe with the highest variance in the mixture data was chosen. Also, we filtered all Affymetrix control probesets, whose probe IDs start with the prefix AFFX-.

GSE11058: The microarray dataset includes samples of the immune cell lines Jurkat, IM-9, Raji, THP-1 and mixtures of them in 4 different proportions [15]. For each proportion there are 3 technical replicates with 24 total samples (12 cell type-specific and 12 mixed samples). We downloaded from GEO the available MicroArray Suite version 5.0 (MAS 5.0) normalized data. With regard to partial and complete semi-supervised methods, a 584 probe signature list defined by CIBERSORT was examined (all tools including CIBERSORT were run with regard to this set based on the used normalized data and processing options). We defined markers as mentioned in previous dataset. In the complete unsupervised mode, all probes that could be mapped to an Entrez identifier based on hgu133plus2.db R/Bioconductor package [16]. Multiple probes per Entrez ID and Affymetrix control probesets were processed as previously.

GSE19380: The microarray dataset includes samples of the rat primary neuronal, astrocytic, oligodendrocytic and microglial cultures (4 biological replicates of each) and mixtures of them in 5 different proportions [1]. For each proportion there are 2 technical replicates with 26 total samples (16 cell type-specific and 10 mixed samples). We downloaded from GEO the available RMA normalized data and unlogged them. Of note, similar to Wang *et al.* [17] we removed the cell type-specific sample 8 (due to poor quality) and all the mixture samples including it (18, 20, 22, 24, 26). We retained sample 25 containing microglial subpopulations but all samples were examined only for three cell types (sample 25 was rescaled to sum to 1 for the three cell types). Notably, results on the full mixed expression dataset are reported for the unsupervised methods. With regard to partial and complete semi-supervised methods, a set of 14 marker probes (for the 3 cell types) extracted from Kuhn *et al.* [1] was used. In the complete unsupervised mode, multiple probes per Entrez ID and Affymetrix control probesets were processed as previously.

Simulated RNA-Seq: We downloaded from DeconRNASeq R/Bioconductor package [10] the in silico mixed RPKM data for 10 samples consisting of brain, muscle, lung, liver and heart tissue and analyzed it with the genome annotation provided. The tool provides also the signature expression matrix for 1570 selected RefSeq IDs and 1520 could be mapped to the mixed data. We defined markers as mentioned in previous datasets. In the complete unsupervised mode, in case of multiple RefSeq IDs per gene name the one with the highest variance across mixed samples was chosen. For Deblender and MMAD in the unsupervised mode, we added an offset of 0.0001 (due to log transformation in certain analysis stages) but also tested an offset of 1.

Synthetic data: To assess the robustness of cluster exemplars for deconvolution, we added Gaussian white noise to the mixture benchmark data with different percentages ($1\%, 5\%, 10\%, 20\%, 30\%, 50\%$). With respect to the percentages $30\%$ and $50\%$, due to negative values appearing in the dataset, we added a constant and since Coefficient of Variation (CV) is sensitive to changes in the mean we adjusted CV cutoffs to allow comparable analysis with the rest noise percentages.

## Patient cancer expression datasets

GSE65135: The microarray dataset includes among others 14 disaggregated lymph node biopsies from patients with follicular lymphoma [11]. These samples were analyzed by flow cytometry to enumerate B cells, CD4 T cells and CD8 T cells. We downloaded from GEO the available expression data normalized with MAS5. In the complete unsupervised mode, multiple probes per Entrez ID and Affymetrix control probesets were processed as previously.

TCGA: We downloaded the level 3 RNASeqV2 RSEM normalized gene counts (with Entrez identifiers) from 1093 breast cancer primary solid tumor samples [18]. An offset of 1 was added to all values prior to analysis. As ground truth estimates, we isolated, from the available biospecimen slide data, the percentages that refer to normal, stromal and tumor cells which along with necrosis summed in most cases close to 1. In case for the same sample both top and bottom slides were available, we averaged the respective percentages. Also, in case the sum was not 1, the proportions for a given sample were scaled accordingly. GO and KEGG pathway enrichment analysis of cluster sets was realized with DAVID tool [19], with whole genome as background.

Molecular subtypes (Luminal A, Luminal B, Her2 (human epidermal growth factor receptor 2) enriched, Basal-like, Normal breast-like) for 840 primary solid samples (matching our dataset) were downloaded from [20] based on the PAM50 subtype annotation [21] for RNA-Seq samples. Tumor purity estimates from four methods and one consensus were downloaded from Aran *et al.* [22]. The data includes results from ESTIMATE [23] that uses gene expression profiles of 141 immune genes and 141 stromal genes; ABSOLUTE [24], using somatic copy-number data; LUMP (leukocytes unmethylation for purity) basing estimates on averaging non-methylated immune-specific CpG sites [22]; IHC applying image analysis on haematoxylin and eosin stain slides produced by the Nationwide Children’s Hospital Biospecimen Core Resource [22]; and the consensus measurement of purity estimations (CPE) representing the median purity level after normalizing results from all methods so that they have equal means and standard deviations [22]. In each method some samples had no available tumor purity results, therefore for pairwise comparison a different set of samples (matched to our dataset) was examined.

## Running settings of methods

All methods were applied on the same normalized expression datasets. In the partial and complete semi-supervised mode, all predefined known signature or cell/tissue type-specific marker probes/genes (as defined by other studies or tools) were present in the imported dataset regardless of our proposed preprocessing (i.e., retaining probes with annotation and selecting one probe per gene identifier) prior to deconvolution. In the complete unsupervised mode the preprocessed datasets were used since that would occur in standard bioinformatics pipelines. With regard to preprocessing, we chose one probe per gene identifier based either on the data of the mixed samples or all samples (i.e., including cell/tissue type-specific expression profiles whenever available) but we selected the first scenario since in a realistic setting the cell/tissue type-specific expression profiles are not available.

With regard to complete semi-supervised NMF-CELLMIX, the preprocessed probes were used as input with all though the predefined known markers included, and we chose to use as initialization of the mixture proportion matrix the DSA estimated result (as proposed in the Cellmix R package). Also, we explored the distance option with Euclidean or Kullback-Leibler divergence as well as the option to scale the NMF result (the best result in terms of correlation with the known mixture proportions is reported). CIBERSORT was run as proposed by the tool disabling though further preprocessing of the data by quantile normalization. DeconRNASeq was run only on the RNA-seq dataset with default settings.

With respect to MMAD, the tool was run only on the semi-supervised mode (with marker gene lists) or the unsupervised with the highly variable probes/genes. We preserved from multiple runs either the best result (when the pool of results differed slightly in terms of correlation performance to the ground truth) or the median result (when the pool of results differed significantly). In all cases the proportion result with the cell/tissue type order configuration that achieved the highest correlation relative to real proportions is reported. We also experimented in the preprocessed datasets with the percentile of most variable genes and applied the $1\%$ (default),$3\%$, $5\%$ and $7\%$. In addition, we tested MMAD* on the microarray datasets in a ‘customized’ setting after preserving genes that displayed across all mixed samples expression values in the range $\left[ 2^{4}-2^{14} \right]$.

Deblender* (over-determined cases) during proportion estimation (S1) was tested with k-means in log space after filtering $10\%$ of the genes with the lowest norm and $10\%$ of the genes with the highest norm and setting $CV\geq0.1$ (for RNA-Seq $CV\geq0.3$). These cutoffs were chosen after experimenting with all datasets and we set them as ‘default’ setting. However, results are also reported if another setting – called ‘strict’, that focuses on detecting the most highly variable genes, performed better after filtering $50\%$ of the genes with the lowest norm and $1\%$ of the genes with the highest norm and $CV\geq0.45$. Deblender* was also tested without any gene filtering in the preprocessed dataset as well as in the non-processed dataset with all probes included. Deblender in S2 both in semi-supervised and unsupervised mode was tested with the proportion result of S1 as initialization in one of the replicated experiments. S2 stage (in unsupervised mode) has submodes that apply either on the complete gene set of the mixture dataset or on the subset of genes closest to exemplars. In semi-supervised mode, we report the result of the first type while in the unsupervised mode of both. In the unsupervised mode, we chose for each cluster the closest $30\%$ genes (cutoff also used in under-determined case) to cluster exemplar and normalized the final $W$ and $H$ (unless stated otherwise); results are reported in case better performance than S1 was achieved. Deblender in under-determined cases was examined with the relevant adapted NMF schemes which in most cases performed better if normalization was applied to the final W and H results. The best performing result is reported, however the performance differences (i.e., correlation with real proportions) among the results were$< 0.2$.

Regarding the TCGA dataset of 1093 primary tumor samples, Deblender* and MMAD* were run with input both the whole gene set (with their default settings used in other benchmark datasets) but also after applying a ‘customized’ gene filtering. In particular, we removed genes with mean normalized RSEM value $< 10$ across samples and genes with zeros in $> 25\%$ of samples. Deblender* was run after including in addition 7 metastasis and 112 normal samples and applying the same ‘customized’ criteria in the combined dataset as aforementioned.

# References

1. Kuhn A, Thu D, Waldvogel HJ, Faull RL, Luthi-Carter R. Population-specific expression analysis (PSEA) reveals molecular changes in diseased brain. Nat Methods. 2011;8: 945-7.
2. Zhong Y, Wan YW, Pang K, Chow LM, Liu Z. Digital sorting of complex tissues for cell type-specific gene expression profiles. BMC Bioinformatics. 2013;14: 89.
3. Parsopoulos KE, Vrahatis MN. Parameter selection and adaptation in Unified Particle Swarm Optimization. Mathematical and Computer Modelling. 2007;46: 198–213.
4. Ahn J, Yuan Y, Parmigiani G, Suraokar MB, Diao L, Wistuba II, Wang W. DeMix: deconvolution for mixed cancer transcriptomes using raw measured data. Bioinformatics. 2013;29: 1865-71.
5. Mohammadi S, Zuckerman N, Goldsmith A, Grama A. A Critical Survey of Deconvolution Methods for Separating cell-types in Complex Tissues. CoRR abs/1510.04583. 2015.
6. Blainey P, Krzywinski M, Altman N. Points of significance: replication. Nat Methods. 2014;11: 879-80.
7. Liebner DA, Huang K, Parvin JD. MMAD: microarray microdissection with analysis of differences is a computational tool for deconvoluting cell type-specific contributions from tissue samples. Bioinformatics. 2014;30: 682-9.
8. Gaujoux R, Seoighe C. CellMix: a comprehensive toolbox for gene expression deconvolution. Bioinformatics. 2013;29: 2211-2.
9. Gaujoux R, Seoighe C. Semi-supervised Nonnegative Matrix Factorization for gene expression deconvolution: a case study. Infect Genet Evol. 2012;12: 913-21
10. Gong T, Szustakowski JD. DeconRNASeq: a statistical framework for deconvolution of heterogeneous tissue samples based on mRNA-Seq data. Bioinformatics. 2013;29: 1083-5.
11. Newman AM, Liu CL, Green MR, Gentles AJ, Feng W, Xu Y, Hoang CD, Diehn M, Alizadeh AA. Robust enumeration of cell subsets from tissue expression profiles. Nat Methods. 2015;12: 453-7.
12. Shen-Orr SS, Tibshirani R, Khatri P, Bodian DL, Staedtler F, Perry NM, Hastie T, Sarwal MM, Davis MM, Butte AJ. Cell type-specific gene expression differences in complex tissues. Nat Methods. 2010;7: 287-9.
13. Gene Expression Omnibus (GEO). Available from: https://www.ncbi.nlm.nih.gov/geo/.
14. Carlson M, 2016. rat2302.db: Affymetrix Rat Genome 230 2.0 Array annotation data (chip rat2302). R/Bioconductor package version 3.2.3.
15. Abbas AR, Wolslegel K, Seshasayee D, Modrusan Z, Clark HF. Deconvolution of blood microarray data identifies cellular activation patterns in systemic lupus erythematosus. PLoS One. 2009;4: e6098.
16. Carlson M, 2016. hgu133plus2.db: Affymetrix Human Genome U133 Plus 2.0 Array annotation data (chip hgu133plus2). R/Bioconductor package version 3.2.3.
17. Wang N, Hoffman EP, Chen L, Chen L, Zhang Z, Liu C, Yu G, Herrington DM, Clarke R, Wang Y. Mathematical modelling of transcriptional heterogeneity identifies novel markers and subpopulations in complex tissues. Sci Rep. 2016;6: 18909.
18. Standardized TCGA data from Broad GDAC Firehose, stddata__2016_01_28 run. [http://gdac.broadinstitute.org/runs/stddata__2016_01_28]. Accessed 12 May 2016.
19. Huang DW, Sherman BT, Lempicki RA. Systematic and integrative analysis of large gene lists using DAVID Bioinformatics Resources. Nature Protoc. 2009;4: 44-57.
20. PAM50 subtypes based on RNA-Seq (TCGA AWG) from UCSC Xena, version 2016-04-27 (http://xena.ucsc.edu/). Accessed 24 December 2016.
21. Parker JS, Mullins M, Cheang MCU, Leung S, Voduc D, Vickery T, Davies S, Fauron C, He X, Hu Z, Quackenbush JF, Stijleman IJ, Palazzo J, Marron JS, Nobel AB, Mardis E, Nielsen TO, Ellis MJ, Perou CM, Bernard PS. Supervised risk predictor of breast cancer based on intrinsic subtypes. J Clin Oncol. 2009;27: 1160–1167.
22. Aran D, Sirota M, Butte AJ. Systematic pan-cancer analysis of tumour purity. Nat Commun. 2015;6: 8971.
23. Yoshihara K, Shahmoradgoli M, Martínez E, Vegesna R, Kim H, Torres-Garcia W, Treviño V, Shen H, Laird PW, Levine DA, Carter SL, Getz G, Stemke-Hale K, Mills GB, Verhaak RG. Inferring tumour purity and stromal and immune cell admixture from expression data. Nat Commun. 2013;4: 2612.
24. Carter SL, Cibulskis K, Helman E, McKenna A, Shen H, Zack T, Laird PW, Onofrio RC, Winckler W, Weir BA, Beroukhim R, Pellman D, Levine DA, Lander ES, Meyerson M, Getz G. Absolute quantification of somatic DNA alterations in human cancer. Nat Biotechnol. 2012;30: 413-21.
